# MiR-21 protected against diabetic cardiomyopathy induced diastolic dysfunction by targeting gelsolin

**DOI:** 10.1186/s12933-018-0767-z

**Published:** 2018-09-04

**Authors:** Beibei Dai, Huaping Li, Jiahui Fan, Yanru Zhao, Zhongwei Yin, Xiang Nie, Dao Wen Wang, Chen Chen

**Affiliations:** 1 Division of Cardiology, Department of Internal Medicine, Tongji Hospital, Tongji Medical College, Huazhong University of Science and Technology, 1095# Jiefang Ave., Wuhan, 430030 China; 2 Hubei Key Laboratory of Genetics and Molecular Mechanisms of Cardiological Disorders, Wuhan, 430030 China

**Keywords:** miRNA, Diabetic cardiomyopathy, Diastolic dysfunction, ROS, NO

## Abstract

**Background:**

Diabetes is a leading cause of mortality and morbidity across the world. Over 50% of deaths among diabetic patients are caused by cardiovascular diseases. Cardiac diastolic dysfunction is one of the key early signs of diabetic cardiomyopathy, which often occurs before systolic dysfunction. However, no drug is currently licensed for its treatment.

**Methods:**

Type 9 adeno-associated virus combined with cardiac Troponin T promoter were employed to manipulate miR-21 expression in the leptin receptor-deficient (db/db) mice. Cardiac structure and functions were measured by echocardiography and hemodynamic examinations. Primary cardiomyocytes and cardiomyocyte cell lines were used to perform gain/loss-of-function assays in vitro.

**Results:**

We observed a significant reduction of miR-21 in the diastolic dysfunctional heart of db/db mice. Remarkably, delivery of miR-21 efficiently protected against the early impairment in cardiac diastolic dysfunction, represented by decreased ROS production, increased bioavailable NO and relieved diabetes-induced cardiomyocyte hypertrophy in db/db mice. Through bioinformatic analysis and Ago2 co-immunoprecipitation, we identified that miR-21 directly targeted gelsolin, a member of the actin-binding proteins, which acted as a transcriptional cofactor in signal transduction. Moreover, down-regulation of gelsolin by siRNA also attenuated the early phase of diabetic cardiomyopathy.

**Conclusion:**

Our findings reveal a new role of miR-21 in attenuating diabetic cardiomyopathy by targeting gelsolin, and provide a molecular basis for developing a miRNA-based therapy against diabetic cardiomyopathy.

**Electronic supplementary material:**

The online version of this article (10.1186/s12933-018-0767-z) contains supplementary material, which is available to authorized users.

## Introduction

Diabetes mellitus is firmly established as a major threat to human health in the twenty-first century due to its alarming rise in incidence over the past two decades, which has attracted considerable attention [1, 2]. Cardiovascular disease is the primary cause of severe morbidity and mortality among the diabetic population. Both experimental and clinical evidences suggest that diabetic subjects are predisposed to a distinct cardiomyopathy, independent of concomitant macro- and microvascular disorders. Diabetic cardiomyopathy is characterized by early impairments in diastolic function, accompanied by the development of cardiac hypertrophy, myocardial fibrosis, and cardiomyocyte apoptosis [3]. However, the pathophysiology of diabetic cardiomyopathy is incompletely understood and currently no specific therapy is available.

Diastolic dysfunction is one of the early key signs of diabetic cardiomyopathy, which often occurs before systolic dysfunction [4–6]. The onset of diabetic cardiomyopathy is characterized by mild cardiac hypertrophy and diastolic dysfunction with preserved systolic function, then leading to heart failure with reduced ejection fraction [7]. A recent study found that compared with heart failure patients without diabetes, diastolic ventricular stiffness was much greater in failing hearts of diabetic patients in the absence of significant coronary artery disease [8]. In addition to impaired systolic function, diabetes and diastolic dysfunction also play important roles in heart failure progress [9, 10]. However, the relationship between the commonly observed metabolic abnormalities and the specific cardiac phenotype is unclear [2]. It was reported that increased myocardial deposition of collagen was responsible for cardiac remodeling and diastolic stiffness in diabetic heart [8, 11]. Myocardial triglyceride accumulation also resulted in diastolic abnormalities [2]. The balance between the generation of ROS and their removal via antioxidant degradation is critical for the maintenance of cardiovascular health. Increased ROS formation also played an integral role in cardiac dysfunction [12, 13]. Decreased NO level contributed to diastolic stiffness [14, 15], while restoration of eNOS-NO signaling ameliorated diabetic heart dysfunction [3, 16]. However, the exact pathogenesis of idiopathic diabetic cardiac dysfunction has yet not been elucidated [3].

MicroRNAs (miRNAs) are a class of endogenous small non-coding RNAs, which emerge as powerful regulators in many essential biological processes [17]. But the roles of miRNAs in diabetes and its cardiovascular complications are not fully discovered [18–20]. It was reported that miR-195 increased ROS production via Sirt1 and Bcl-2, and inhibition of miR-195 may be a promising therapeutic strategy for lipotoxic cardiomyopathy [21]. miR-30d promoted cardiomyocyte pyroptosis in diabetic cardiomyopathy [22]. Kuwabara et al. revealed that high-fat diet-induced cardiac hypertrophy was ameliorated in cardiomyocyte-specific miR-451 knockout mice [23]. Similarly, we found that miR-30c was crucial in diabetic cardiomyopathy by regulating autophagy [24]. Circulating miRNAs are considered as potential biomarkers of various diseases [25]. Decreased plasma level of miR-21 was found in patients with diabetes in large population-based cohorts [26–28]. Recently, it was found that miRNAs mediated the benefits of exercise and bariatric surgery to the heart in diabetic/prediabetic patients, and the decreased circulating miR-21 were restored after bariatric surgery or physical activity [25, 29]. Moreover, upregulation of miR-21 or downregulation of its targets could lead to diabetic cardiac dysfunction [30–32]. Therefore, miR-21 might play important roles in diabetic cardiomyopathy, and it may be a new therapeutic target for metabolic diseases such as T2DM and obesity. However, the roles of miR-21 in cardiac diseases are controversial. Thomas et al. showed that miR-21 promoted cardiac fibroblast survival, which led to fibrosis, hypertrophy and cardiac dysfunction [33], whereas Cheng et al. found that miR-21 protected against the H_2_O_2_-induced damage in cardiac myocytes via PDCD4 and AP-1 pathway [34]. A study reported that high glucose increased the expression level of miR-21 in fibroblasts from heart, and miR-21 promoted collagen synthesis in vitro [35]. Recent studies found that miR-21 inhibition significantly decreased body weight, adipocyte size and serum triglycerides, on the other hand, miR-21 reversed high glucose and high insulin induced IR in 3T3-L1 adipocytes, possibly through modulating the PTEN-AKT pathway [31, 36]. It was supposed that the same miRNA from different cell sources may exert various effects on the same disease [25, 37, 38]. Previously, we revealed a positive function of miR-21 in mitochondrial translation, which was sufficient to reduce blood pressure and alleviate cardiac hypertrophy in spontaneous hypertension rat [39]. By now, not only the role of miR-21 in diabetic cardiomyopathy is unclear, but also the predominate functional cell type in diabetic cardiomyopathy remains unknown.

In the present study, we investigated the roles of miR-21 in diastolic dysfunction of early diabetic cardiomyopathy in vitro and in vivo, and found that miR-21 protected against diastolic dysfunction and cardiac hypertrophy, by reducing ROS production via gelsolin in db/db mice, which suggested a new therapeutic strategy against diabetic cardiomyopathy.

## Research design and methods

### Cell culture and transfection

H9c2 and HEK293 cells from ATCC (Manassas, VA) were maintained in H-DMEM supplemented with 10% FBS at 37 °C with a 95% air, 5% CO_2_ atmosphere. Cells were incubated with normal (5 mM) or high (33 mM) glucose for 48 h and then collected. For high fatty acid (FA) stimulation, H9c2 cells were treated with palmitate (250 μM) for 48 h, as previously described [24].

HL-1 cell line, which is a cardiac muscle cell line derived from the AT-1 mouse atrial cardiomyocyte tumor lineage was also from ATCC (Manassas, VA). And cells were cultured in Claycomb Medium (Sigma, Shanghai, China) with 10% FBS, 4 mM l-gutamine (Sigma, Shanghai, China) and 100 μM norepinephrine (Sigma, Shanghai, China) at 37 °C with a 95% air, 5% CO_2_ atmosphere as previously described [40].

Human cardiomyocytes were purchased from Sciencell (San Diego, CA, Catalog Number: 6200). Cells were carefully cultured according to product instruction.

MiRNA mimics, miRNA inhibitor, siRNAs and relative controls were purchased from RiboBio (Guangzhou, China). Transfection with miR-21 mimics (100 nM), siRNAs (100 nM), and relative controls (100 nM) was performed with Lipofectamine 2000 (Invitrogen, Carlsbad, CA), according to the manufacturer’s recommendations.

### Isolation of primary cardiomyocytes

Cardiomyocytes (CMs) and non-cardiomyocytes (NCMs) were isolated from adult mice, as described previously [41]. Firstly, we perfused and digested the left ventricles of hearts, and then dissected them into small pieces for dissociation in transfer buffer. Secondly, we filtered the cell solution and the cells were settled by sedimentation for several minutes in Falcon tubes. After that, we transferred cell pellets and supernatants to other Falcon tubes for further separation. Cell pellets were re-suspended in transfer buffer and settled by repeated precipitation for twice. After a second precipitation, the cell pellets were examined for typical rod-shaped cell morphology before RNA extraction to verify the expression of CM markers. The initial supernatant was first centrifuged at 50*g* (3 min) and then at 300*g* (5 min) before verification of the pelleted cell NCM identity by qRT-PCR assessment of fibroblast and CM markers (Additional file 1: Fig. S1) [42].

### FFA preparation

FFA solutions were prepared as described previously [43]. Briefly, 100 mM palmitate (Sigma, P9767, Shanghai, China) stocks were prepared in 0.1 M NaOH at 70 °C and filtered. Twenty percent (weight/volume) palmitate-free BSA (Sigma, A2153, Shanghai, China) solution was prepared in serum-free DMEM. After the palmitate dissolved, the palmitate solutions were added to serum-free DMEM containing BSA. The 25 mM palmitate/20% BSA solution was prepared by complexing appropriate amounts of palmitate to 20% BSA in a 40 °C water bath.

### Western blotting

Protein concentrations were determined by the BCA method. For Western blotting, total cell lysate was resolved by SDS-PAGE, transferred to PVDF membrane, and blocked with 5% non-fat dry milk in TBS-T. The membrane was incubated with primary antibody overnight at 4 °C, followed by peroxidase-conjugated secondary antibody for 2 h, and finally developed with the ECL system (Beyotime Institute of Biotechnology, Nanjing, China). Antibodies against phospho-eNOS (Ser1177) (Catalog No: 9570s), phospho-eNOS (Thr495) (Catalog No: 9574s), and eNOS (Catalog No: 32027s) were purchased from Cell Signaling Technology (Shanghai, China). Anti-GAPDH was from Tianjin Sungene Biotech Co., Ltd. (Tianjin, China) (Catalog No: KM9002T). Anti-Akt1/2/3 (Ser473) (Catalog No: sc-8312) and anti-p-Akt1/2/3 (Ser473) (Catalog No: sc-7985-R) were from Santa Cruz Biotech (Santa Cruz, CA). Anti-gelsolin (Catalog No: ab134183) was from Abcam (Shanghai, China). Western blotting results were quantified by densitometry and processed with the ImageJ software (National Institutes of Health software).

### RNA isolation and detection

Total RNA was collected from frozen tissues or cells by TRIzol Reagent (Invitrogen, Carlsbad, CA) according to the manufacturer’s protocol. Then, total RNA (2 μg) was reverse transcribed using the first-strand cDNA synthesis kit (Thermo Scientific, Carlsbad, CA). Amplification and detection of specific products were performed on a 7900HT Fast Real-Time PCR system (Applied Biosystems, Foster City, CA). The Bulge-LoopTM miRNA qPCR Primer Sets (RiboBio, Guangzhou, China) were used to detect miRNA expression. The primers of gene PCR were synthesized by Tianyihuiyuan (Wuhan, China) were used to access mRNA expression levels. U6 was used as an internal control for miRNA template normalization, and GADPH was used for mRNA template normalization. The sequences of the primers used were listed in Additional file 1: Table S1. The qRT-PCR reactions were run in triplicate, and the signal was collected at the end of every cycle. The relative gene expression was calculated by comparing cycle times (Ct) for each target PCR as previously described [44]. The qRT-PCR productions were collected for sequencing to validate the specific of gene primers.

### RNA immunoprecipitation

Twenty-four hours after transfection with miR-21 mimics or miRNA random control, cells were lysed and then immunoprecipitated with anti-Ago2 antibody (Abnova Corporation, Taiwan, China) or IgG (Abclonal, Wuhan, China) using protein A/G magnetic beads (Thermo Scientific, Shanghai, China), as described [45]. After incubating the cells overnight at 4 °C, 40 μL of protein A/G magnetic beads were added, and the solution was incubated for 2 h at 4 °C. The beads were washed five times with PBS and resuspended in 60 μL Laemmli buffer. The remaining products were extracted with TRIzol, and the levels of mRNA were quantified by real-time PCR.

### Construction of rAAV

To manipulate the expression of miR-21 in vivo, the rAAV (type 9) combined with cardiac troponin T (cTnT) promoter was employed. The rAAV system (type 9) was a kind gift from Dr. Xiao Xiao (University of North Carolina at Chapel Hill). For construction of the adenoviruses, oligonucleotides were designed as miR-21, (5-TGCACTGCAGTAGCTTATCAGACTGATGTTGATTCAAGAGATCAACATCAGTCTGATAAGCTACCATGGCATG-3), miR-21-TUD, (5-GACGGCGCTAGGATCATCAACATCGAATAGTTCTACTGACTACAACTACAAGTATTCTGGTCACAGAATACAACATCGAATAGTTCTACTGACTACAACTACAAGATGATCCTAGCGCCGTC-3) according to the mature sequence of hsa-miR-21 provided by miRBase (Accession: MIMAT0000076). Then, the oligonucleotides were synthesis and inserted into pAAV vector at PstI and NcoI sites. These vectors were named pAAV-tnt-miR-21 and pAAV-tnt-miR-21-TUD, respectively. The constructs were sequenced to confirm the DNA sequences. The designed gene plasmid, pXX9, and pHelper were packaged together by triple plasmids co-transfection in HEK293 cells, and then purified as described previously [46]. The resultant rAAVs were assigned as rAAV-GFP, rAAV-tnt-miR-21, rAAV-tnt-miR-21-TUD, and rAAV-tnt-GFP, respectively. The specificity of cardiac delivery was determined by GFP detection (Additional file 1: Fig. S2).

### Animals

All experiments were performed with the approval of the Animal Research Committee of Tongji Medical College, and in accordance with ARRIVE and NIH guidelines for animal welfare. For in vivo experiments, male db/db mice on C57BL/Ks background and control C57BL/Ks mice (Model Animal Research Center of Nanjing University, Nanjing, China) were used. All the animals were maintained with 12-h light/12-h dark photoperiods with free access to water and food. We randomly divided db/db mice into four groups (control, rAAV-tnt-GFP, rAAV-tnt-miR-21, and rAAV-tnt-miR-21-TUD, n ≥ 8 each group). They were injected with corresponding rAAVs via tail vein at the age of 8 weeks. All surgery was performed under sodium pentobarbital anesthesia to minimize suffering. Through intraperitoneal injections of a ketamine (80 mg/kg) and xylazine (5 mg/kg) mixture, anaesthetization of mice was performed. To assess the adequacy of anesthesia during hemodynamic examinations, parameters such as responsiveness, blood pressure, respiratory and heart rates were monitored. Then they were sacrificed by CO_2_ inhalation after the surgical procedures. The rAAV-treated db/db and control C57BL/Ks mice were sacrificed at 20 weeks and tissue samples were snap-frozen in liquid nitrogen or collected for paraffin embedding.

### Hemodynamic analyses

Mouse hemodynamics were assessed as previously described [47]. After anaesthetization, left ventricular (LV) catheterization was performed on mice before sacrifice. The portion of the right carotid artery next to the trachea was isolated from the surrounding tissue and nerves. A 1.0 Fr Millar Mikrotip Catheter Transducer (Millar 1.4F, SPR 835, Millar Instruments, Houston, TX) connected to a pressure transducer (Millar Instruments, Houston, TX) was inserted through the right carotid artery into the LV cavity. Hemodynamic parameters were recorded and analyzed with PowerLab/8sp and LabChart 7.2.1 software. PVAN software (Millar Instruments, Houston, TX) was used to perform the cardiac pressure–volume analysis. The peak instantaneous rate of the LV pressure increase and decline (dp/dt max, dp/dt min) were measured. All data were averages of at least five measurements, each measurement concluded at least ten successive loops.

### Echocardiography

After anesthetization, echocardiographic analysis was performed to determine cardiac function of 20-week-old mice using a high-resolution imaging system with a 30-MHz high frequency scanhead (VisualSonics Vevo770, VisualSonics, Toronto, Canada), as described previously [24]. Briefly, a parasternal long-axis B-mode image was acquired with appropriate positioning of the scan head, so that the maximum LV length could be identified. Then a clockwise 90 rotation at the papillary muscle level depicted the parasternal short-axis view. An M-mode cursor was positioned perpendicular to the anterior and posterior walls of the left ventricle from this view and M-mode image loops were obtained for measurement of wall thickness and chamber dimensions. Each of these captured image loops included 11–20 cardiac cycles, and data were averages from at least three cycles per loop.

LVEF was calculated as follows: LVEF = (left ventricular end-diastolic volume [LVEDV] − left ventricular end-systolic volume [LVESV])/LVEDV × 100%. FS was calculated as follows: FS % = (left ventricular end diastolic dimension [LVDd] − left ventricular end systolic dimensions [LVDs])/LVDd × 100%.

### Histological analysis

Formalin-fixed hearts were embedded in paraffin and sectioned into 4 mm slices. The morphology was detected by H&E staining. Oil Red O staining was applied to 7 μm frozen sections. Lipid deposition were visualized by microscope, and measured by Image-Pro Plus Version 6.0 (Media Cybernetics, Bethesda, MD). The oxidative fluorescent dye, dihydroethidium (DHE; Invitrogen, Carlsbad, CA) was applied to 7 μm frozen sections from hearts at 40 μmol for 30 min. Fluorescence intensity was measured under a Nikon DXM1200 fluorescence microscope and images were analyzed with the Image-Pro software (Media Cybernetics, Bethesda, MD). Total ROS was quantified using 2,7-dichlorodihydrofluorescein diacetate (DCFH-DA, Invitrogen, Carlsbad, CA). TUNEL staining and Sirius Red Staining were also applied to detect apoptotic cells and myocardial fibrosis in hearts.

### Biochemistry examinations

Biochemical parameters TC, TG, LDL, and HDL in plasma, TC and TG in heart or H9c2 cells were detected by GRO-PAP method (Nanjing Jiancheng Bioengineering Institute, Nanjing, China). After mice were fasted overnight, blood glucose level was measured by Glucose LiquiColor Test (Stanbio Laboratory, Boerne, TX) every 4 weeks.

### Measurements of NO production

The concentration of NO was measured as nitrite (the final stable state of NO) accumulation in the culture medium using a NO assay kit (Beyotime, Nanjing, China) according to the manufacture’s instruction. Briefly, 50 μL of supernatant was subjected to colorimetricreaction with Griess reagents. After reaction, the NO concentration was quantified by absorbance at 540 nm using a microplate reader (BIO-TEK Instruments, Shanghai, China).

### Dual luciferase assay

First, we transfected 400 ng of pMIR-GSN 3′ UTR, pMIR-GSN 3′ UTR mutant, or the empty vector into HEK293 cells with 40 ng of pRL-TK plasmid (Promega, Madison, WI), respectively. Then, miR-21 mimics or miR-con was co-transfected with those reporter plasmids at a final concentration of 100 nM. Forty-eight hours later, luciferase activity was detected by Dual-Luciferase Reporter Assay System (Promega, Beijing, China) according to the manufacturer’s protocol. Renilla luciferase activity was used to normalize the transfection efficiency.

### Statistics

All data are analyzed using Prism 5 (GraphPad Software, San Diego, CA) and presented as mean ± SEM. The Student’s t test and ANOVA were performed to determine statistically significant differences among treated groups, as appropriate. In all cases, a value of p < 0.05 was considered to be statistically significant.

## Results

### Overexpression of miR-21 protects against diastolic dysfunction in db/db mice

**Fig. 1:**
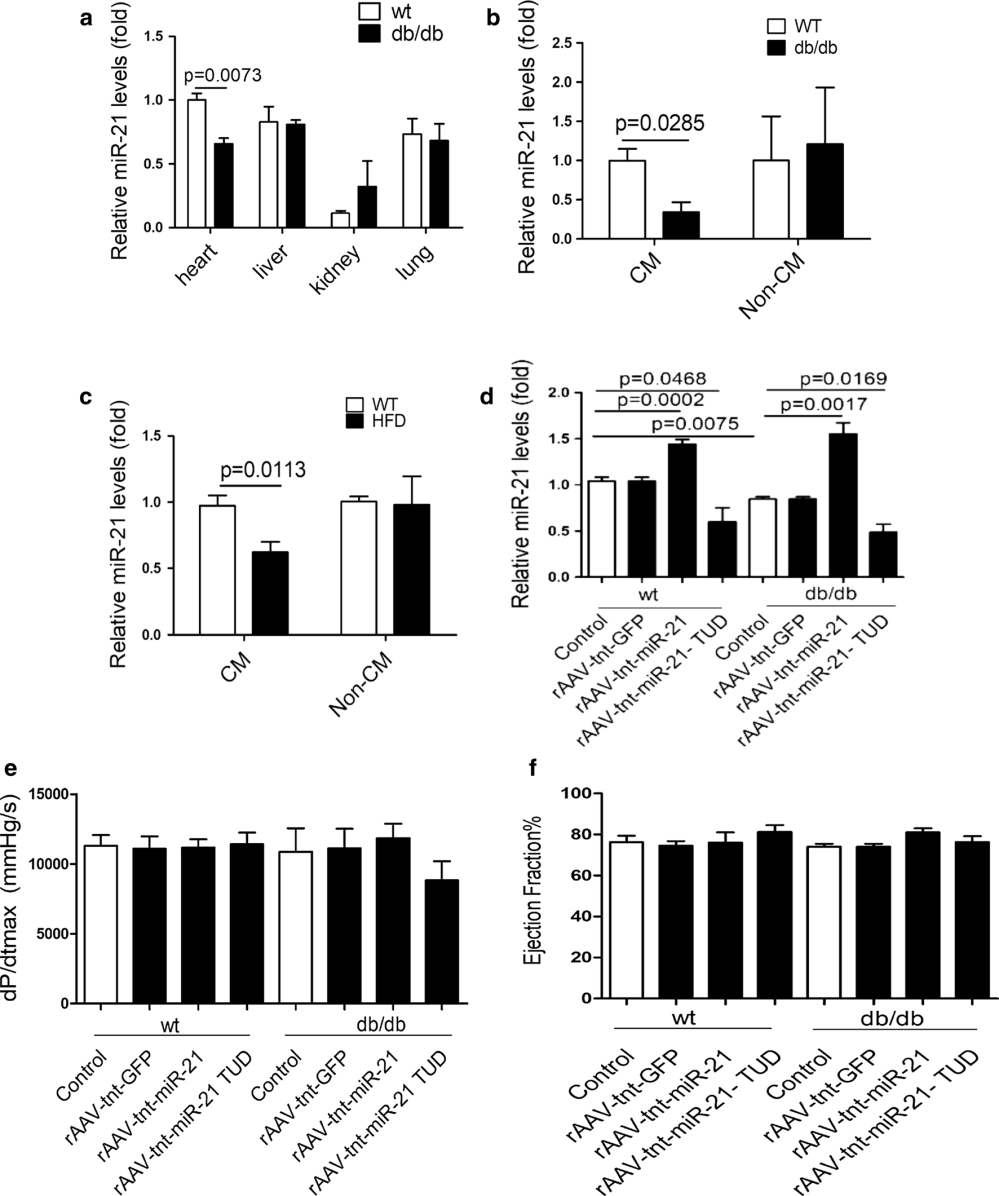
Overexpression of miR-21 protects against diastolic dysfunction in db/db mice. **a** Relative expression of miR-21 among different organs. **b** Relative expression of miR-21 in isolated CMs and NCMs from db/db mice. **c** Relative expression of miR-21 in isolated CMs and NCMs from high fat diet fed mice. **d** Relative expression of miR-21 in heart from treated mice. **e**  Hemodynamic analysis of db/db mice and C57BL/Ks controls. dp/dt_max_, peak instantaneous rate of LV pressure increase. **f**  Echocardiographic analysis of db/db mice and controls. EF% (ejection fraction) was quantitatively analyzed

To explore the roles of miR-21 in the early phase of diabetic cardiomyopathy, real-time PCR was used to detect miR-21 expression in 20-week-old db/db mice, a well-established animal model of diabetic cardiomyopathy [48]. Compared with wild type controls, significantly decreased miR-21 was observed in heart of db/db mice (Fig. 1a). Interestingly, miR-21 was specifically down-regulated in cardiomyocytes of db/db mice (Fig. 1b). Similarly, high fat diet-induced diabetic mice also showed a significant decrease of miR-21 expression in cardiomyocytes as db/db mice (Fig. 1c).

**Fig. 2:**
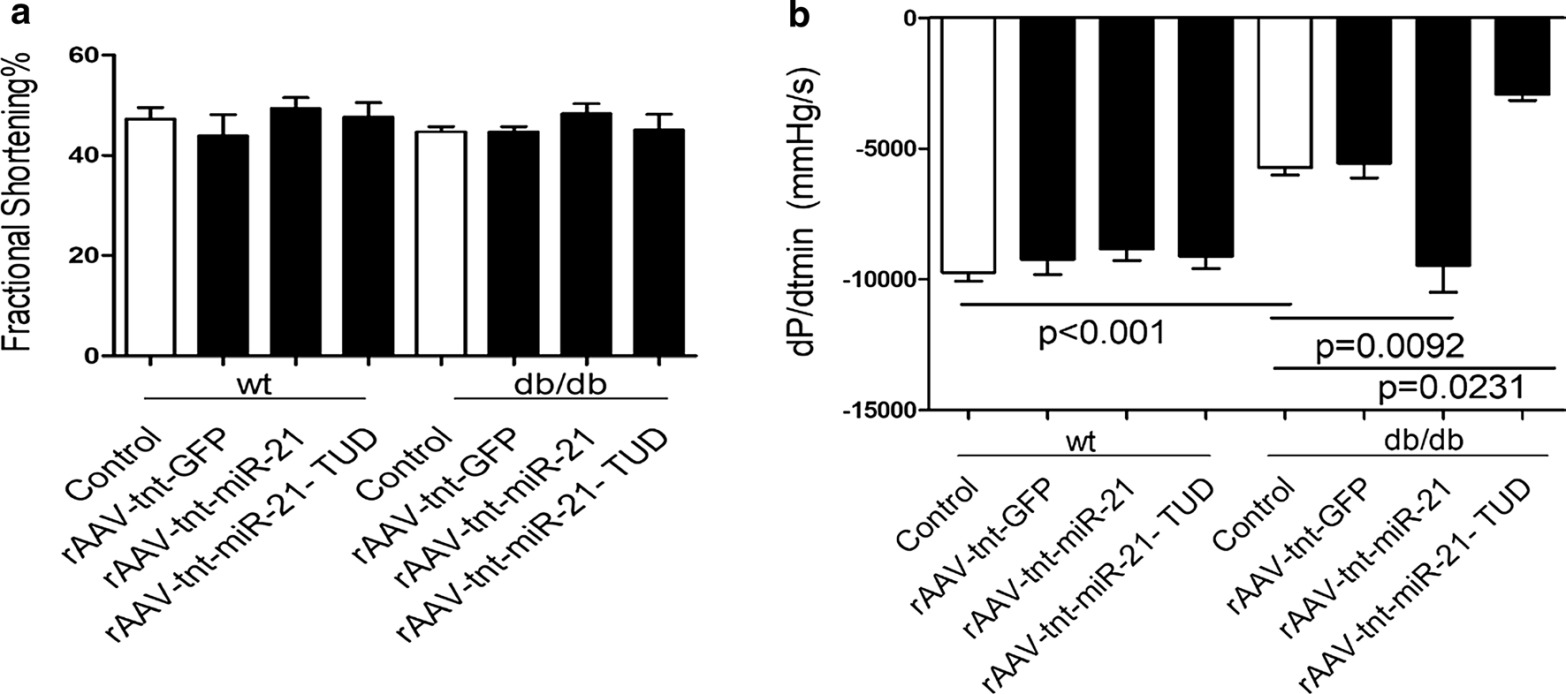
Overexpression of miR-21 protects against diastolic dysfunction in db/db mice. **a** Echocardiographic analysis of db/db mice and controls. FS% (fractional shortening) was quantitatively analyzed. **b** Hemodynamic analysis of db/db mice and C57BL/Ks controls. dp/dt_min_, peak instantaneous rate of LV pressure increase decline

Next, we employed the db/db mice model to investigate the roles of myocardial miR-21 in the early phase of diabetic cardiomyopathy by using rAAV combined with cTnT promoter delivery system. Compared with wild type controls, miR-21 was decreased in db/db mice heart, and cardiac miR-21 level was increased in rAAV-tnt-miR-21 treated mice, while rAAV-tnt-miR-21-TUD (tough decoy) treatment exerted opposite effects (Fig. 1d). As shown in Fig. 1e, f, 2a and Additional file 1: Fig. S3A–G, there was no significant change in systolic function among the control and treated mice, which was consistent with previous data [4–6]. However, miR-21 overexpression rescued diastolic dysfunction in db/db mice (Fig. 2b, Additional file 1: Fig. S3H). Moreover, blood glucose, body weight, LDL, TG, and TC levels were increased in db/db mice, but cardiac overexpression or inhibition of miR-21 had no effects on these metabolic characteristics (Additional file 1: Fig. S4).

Our data indicated that cardiac specific overexpression of miR-21 protected against diastolic dysfunction, which was the key early phase sign of diabetic cardiomyopathy in db/db mice, independent of systemic metabolic improvements, such as blood glucose and lipid levels.

### MiR-21 protects against cardiac hypertrophy in diastolic dysfunction db/db mice

**Fig. 3:**
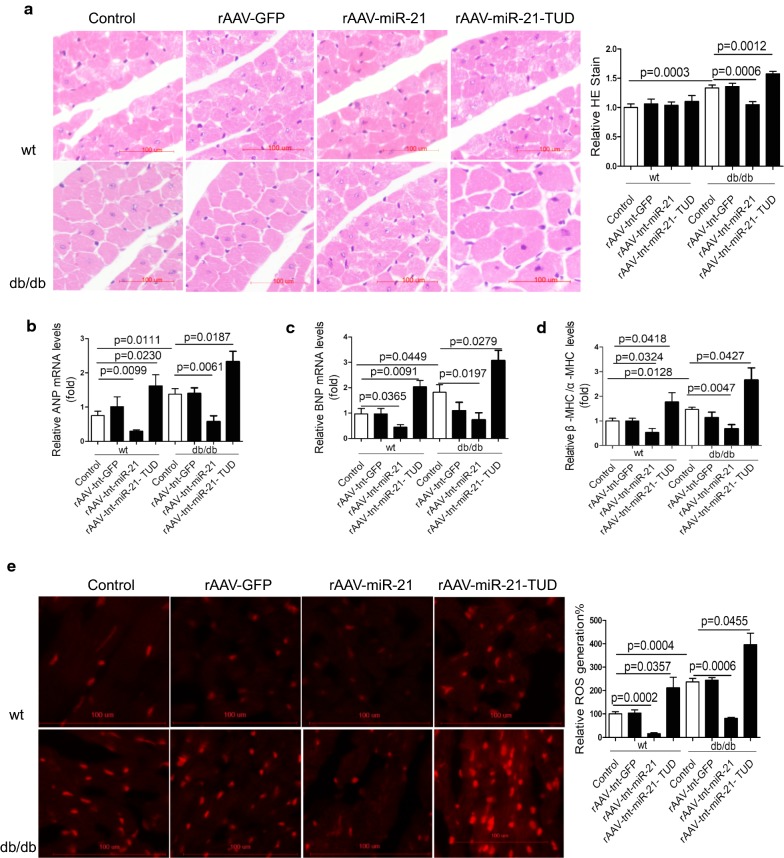
MiR-21 protects against cardiac hypertrophy in diastolic dysfunction db/db mice. **a** Histological analysis of cardiomyocyte by H&E staining. **b**–**d** Relative expression levels of ANP, BNP, β-MHC/α-MHC in heart from treated mice. **e** Representative images of ROS detected by DHE probe in frozen heart sections of db/db mice compared to controls

To further investigate the mechanisms underlying diastolic dysfunction, we found that cardiac hypertrophy was increased in db/db mice compared with wild type controls (Fig. 3a). Consistently, overexpression of miR-21 significantly decreased while inhibition of miR-21 increased cardiac hypertrophy in db/db mice (Fig. 3a). Moreover, the expression of hypertrophy markers, such as ANP, BNP, and β-/α-MHC ratio showed a similar change in treated db/db mice (Fig. 3b–d). However, there was neither fibrosis nor apoptosis among all groups (Additional file 1: Fig. S5A, B). Although lipid deposition measured by Oil Red was increased in db/db mice heart compared to controls, miR-21 had no effect on lipid deposition in db/db mice heart (Additional file 1: Fig. S5C, D). These data suggested that miR-21 did not participate in diastolic dysfunction via regulating fibrosis, apoptosis or lipid accumulation in db/db mice.

**Fig. 4:**
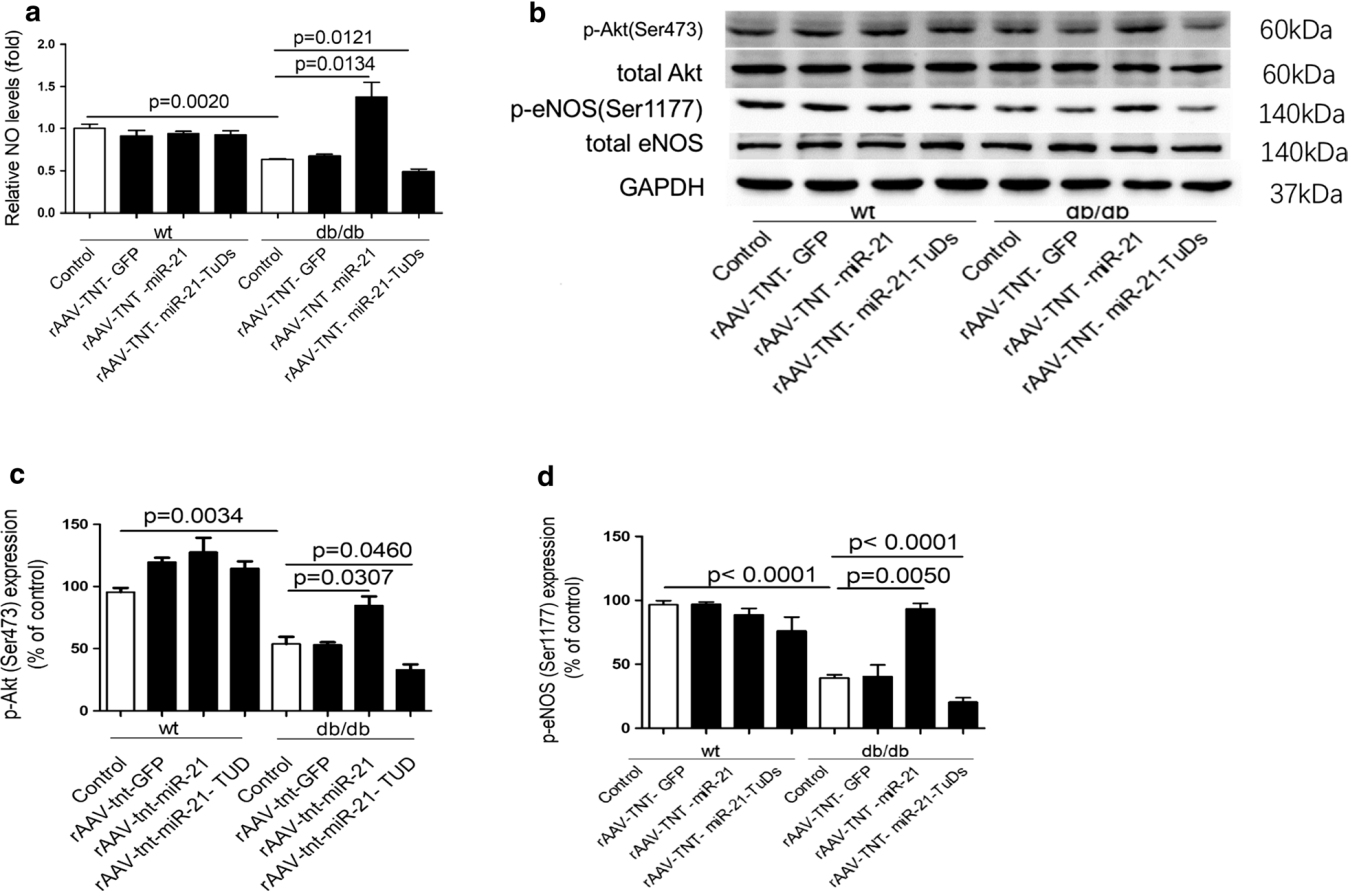
MiR-21 protects against cardiac hypertrophy in diastolic dysfunction db/db mice. **a** Relative NO levels in heart from treated mice. **b**–**c** Protein levels of p-Akt(Ser473) and p-eNOS(Ser1177) in heart from treated mice

It was reported that ROS may contribute to the initial cardiac diastolic dysfunction, while increasing NO bioavailability maybe beneficial for recovering impaired diastolic function in diabetes [12–15, 49]. Interestingly, we found elevated cardiac ROS levels in db/db mice in comparison with wild type controls, and overexpression of miR-21 decreased ROS levels in db/db mice (Fig. 3e). Meanwhile, we observed reduced NO level in the heart of db/db mice, and miR-21 increased while miR-21-TUD decreased NO level in db/db heart (Fig. 4a). It was reported that increased phosphorylation of Akt at Ser473 and eNOS at Ser1177 may improve NO release and cardiac function [16, 50]. Consistently, miR-21 increased the expression of p-Akt(Ser473) and p-eNOS(Ser1177) in db/db mice (Fig. 4b–d). In wild type mice, the NO level, expression of p-Akt(Ser473) and p-eNOS(Ser1177) were not changed by miR-21 (Fig. 4a–d). Meanwhile, we found that the expression of p-eNOS(Thr495) and iNOS were not affected by miR-21 in both wild type mice and db/db mice (Additional file 1: Fig. S6).

In conclusion, our data suggested that miR-21 might protect against diastolic dysfunction by inhibiting cardiac hypertrophy via decreasing ROS level and increasing eNOS induced-NO release in db/db mice.

### MiR-21 attenuates diabetic condition-induced cardiomyocyte hypertrophy in vitro

**Fig. 5:**
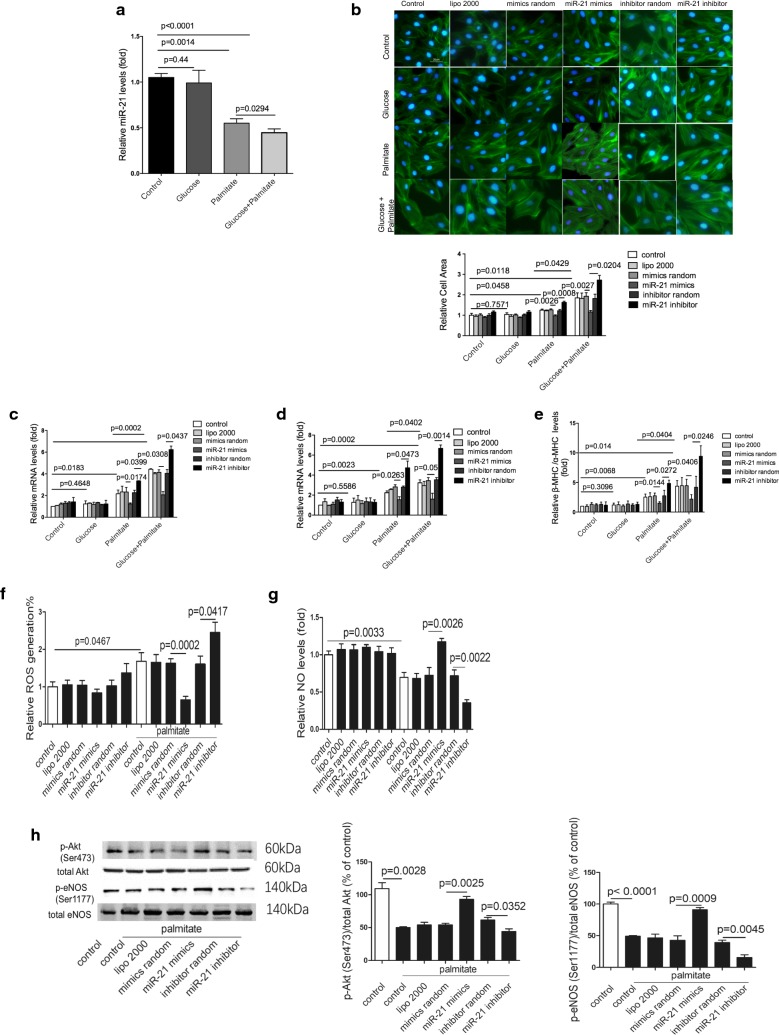
MiR-21 attenuates diabetic condition-induced cardiomyocyte hypertrophy in vitro. **a** Relative expression of miR-21 in treated H9c2 cells. **b** Representative images and relative cell area determined by quantitation analysis of cardiomyocyte by Phalloidin staining in treated H9c2 cells. **c**–**e** Relative expression levels of ANP, BNP, and β-MHC/α-MHC in treated H9c2 cells. **f** ROS generation in treated H9c2 cells. **g** Relative NO levels in treated H9c2 cells. **h** Protein levels of p-Akt(Ser473) and p-eNOS(Ser 1177) in treated H9c2 cells

To further determine whether the decreased miR-21 was hyperglycemia or hyperlipidemia-dependent, cultured H9c2 cells were treated with high glucose, palmitate, palmitate plus glucose, respectively. Interestingly, palmitate but not glucose treatment significantly decreased miR-21 expression in H9c2 cells. Palmitate plus glucose together further suppressed the palmitate-decreased miR-21 expression (Fig. 5a). Moreover, hypertrophy observed in palmitate-treated cells was significantly attenuated by miR-21 mimics treatment (Fig. 5b). Further, miR-21 decreased the expression of cardiac hypertrophy markers in palmitate-treated cells (Fig. 5c–e). All effects were enhanced by treatment of palmitate plus glucose together, comparing with palmitate alone (Fig. 5a–e). More importantly, palmitate treatment induced ROS and decreased NO level in cardiomyocytes were attenuated by miR-21 mimics transfection (Fig. 5f, g). Consistently, miR-21 increased the phosphorylation of Akt at Ser473 and eNOS at Ser1177 (Fig. 5h). Still, the expression levels of p-eNOS(Thr495) and iNOS were not changed by miR-21 (Additional file 1: Fig. S7). Additionally, in physiological status, miR-21 had neither effect on cardiomyocyte area, ROS and NO levels, nor p-eNOS and iNOS expression levels (Fig. 5b–g, Additional file 1: Fig. S8). Experiments in human cardiac myocytes also showed similar results (Additional file 1: Fig. S9).

These data indicated that miR-21 attenuated cardiomyocyte hypertrophy via decreasing ROS level and increasing NO production in palmitate treated-cardiomyocyte.

### Gelsolin is a direct target of miR-21

**Fig. 6:**
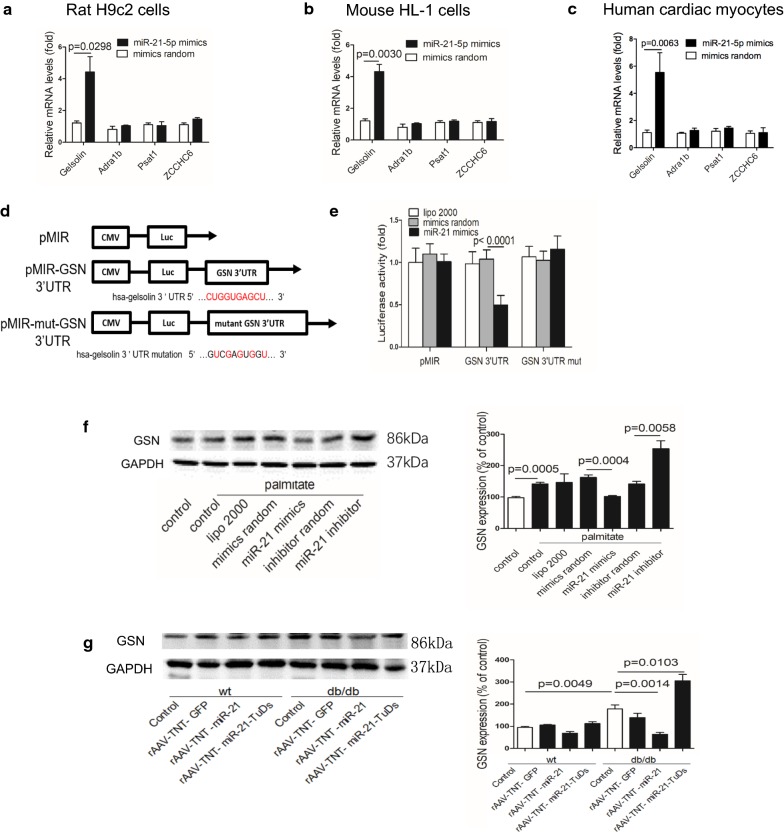
Gelsolin is a direct target of miR-21. **a** Relative mRNA levels detected by Ago2-RIP in treated H9c2 cells. **b** Relative mRNA levels detected by Ago2-RIP in treated HL-1 cells. **c** Relative mRNA levels detected by Ago2-RIP in treated human cardiac myocytes. **d** Schematic diagram of the luciferase reporter plasmids. **e** Regulation of miR-21 on 3′ UTR of GSN in HEK293 cells detected by luciferase reporter assays. **f** Protein levels of GSN in treated H9c2 cells. **g** Protein levels of GSN in treated mice

We predicted and evaluated the potential targets of miR-21 in cultured cells. As reported previously [51], we performed mRNA-microarray in diabetic cardiomyopathy (db/db mice) heart, and found that 69 genes were upregulated in db/db heart (Additional file 1: Table S2). Then bioinformatic analysis was performed to identify the binding potential of miR-21 to those dysregulated genes using the BiBiServ Tool. As a result, 4 genes (Gelsolin, Adra1b, Psat1 and ZCCHC6) were predicted to be potential targets of miR-21 (Additional file 1: Fig. S10). Secondly, we performed RNA co-immunoprecipitation with Ago2 antibody, and found increased association of gelsolin (GSN) mRNA with Ago2 after miR-21 treatment in rat H9c2 cells, mouse HL-1 cells and human cardiac myocytes (Fig. 6a–c). Next, we cloned the 3′ UTR of human GSN (including wild-type and seed region mutated sequence) to pMIR-report vector, respectively (Fig. 6d). Results showed that after co-transfecting with miR-21 mimics, the relative luciferase activity of pMIR-GSN 3′ UTR in HEK293 cells was significantly suppressed compared with mimics random. However, this suppressive effect of miR-21 was abolished by mutating GSN 3′ UTR (Fig. 6e). Finally, Western blots showed a higher expression of GSN in cultured H9c2 cells exposed to palmitate compared with control, and miR-21 transfection decreased the enhanced GSN level, while miR-21 inhibitor further increased the GSN expression (Fig. 6f). Further, western blot showed that cardiac GSN was increased in db/db mice compared with wild type controls, and miR-21 treatment significantly reduced GSN expression in db/db mice (Fig. 6g). These data indicated that miR-21 directly targeted GSN in vitro and in vivo.

### MiR-21 alleviates palmitate-induced injury via GSN

**Fig. 7:**
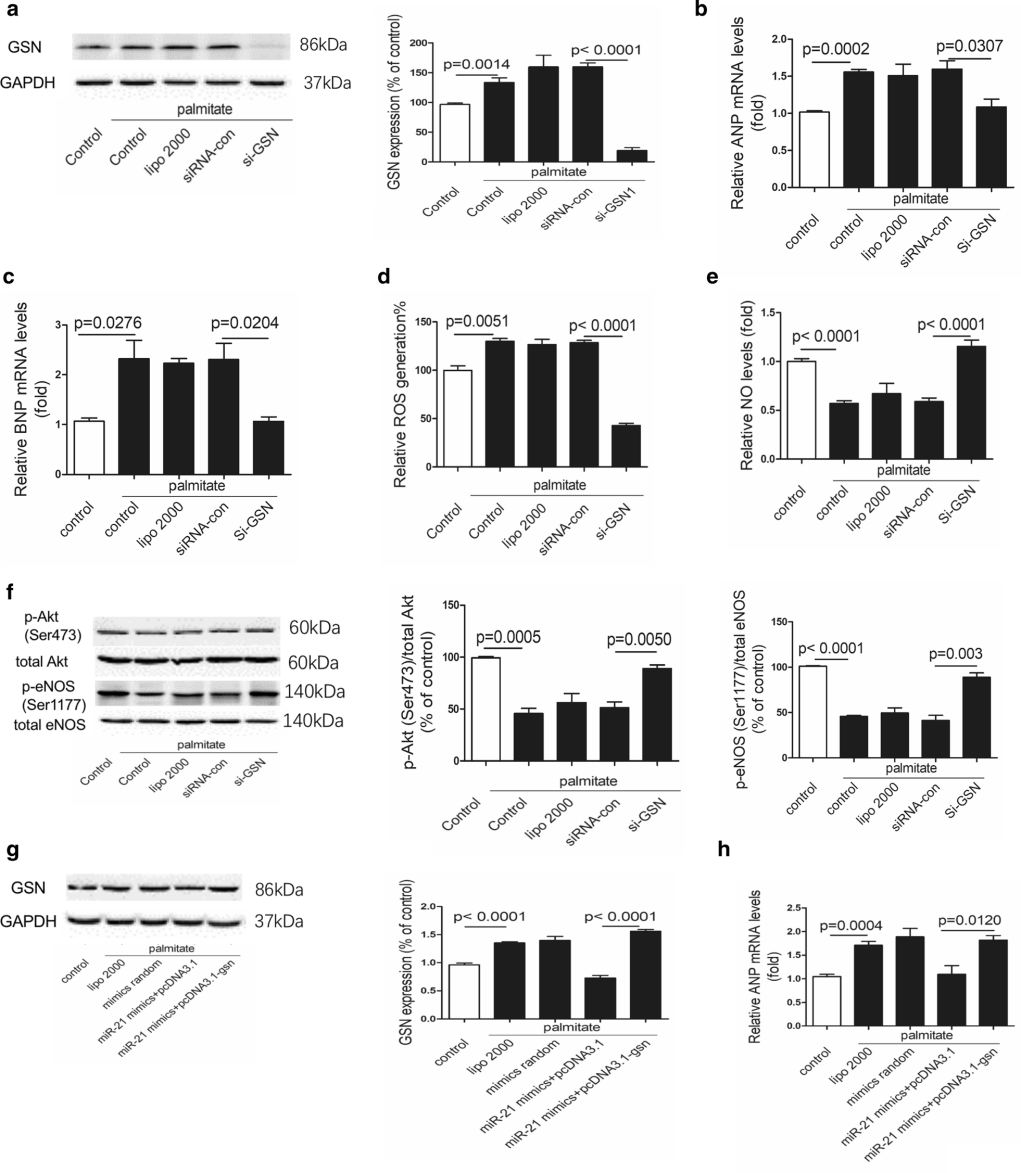
MiR-21 alleviates palmitate-induced injury via GSN. **a** Protein levels of GSN in treated H9c2 cells. **b**, **c** Relative expression levels of ANP and BNP in treated H9c2 cells. **d** ROS generation in treated H9c2 cells. **e** Relative NO levels in treated H9c2 cells. **f** Protein levels of p-Akt(Ser473) and p-eNOS(Ser1177) in treated H9c2 cells. **g** Protein levels of GSN in treated H9c2 cells. **h** Relative expression levels of ANP in treated H9c2 cells

Consistent with the effects of miR-21, down-regulation of GSN by siRNA decreased hypertrophy markers expression and ROS level, but increased NO production and expression of p-Akt(Ser473) and p-eNOS(Ser1177) in palmitate treated cells (Fig. 7a–f).

**Fig. 8:**
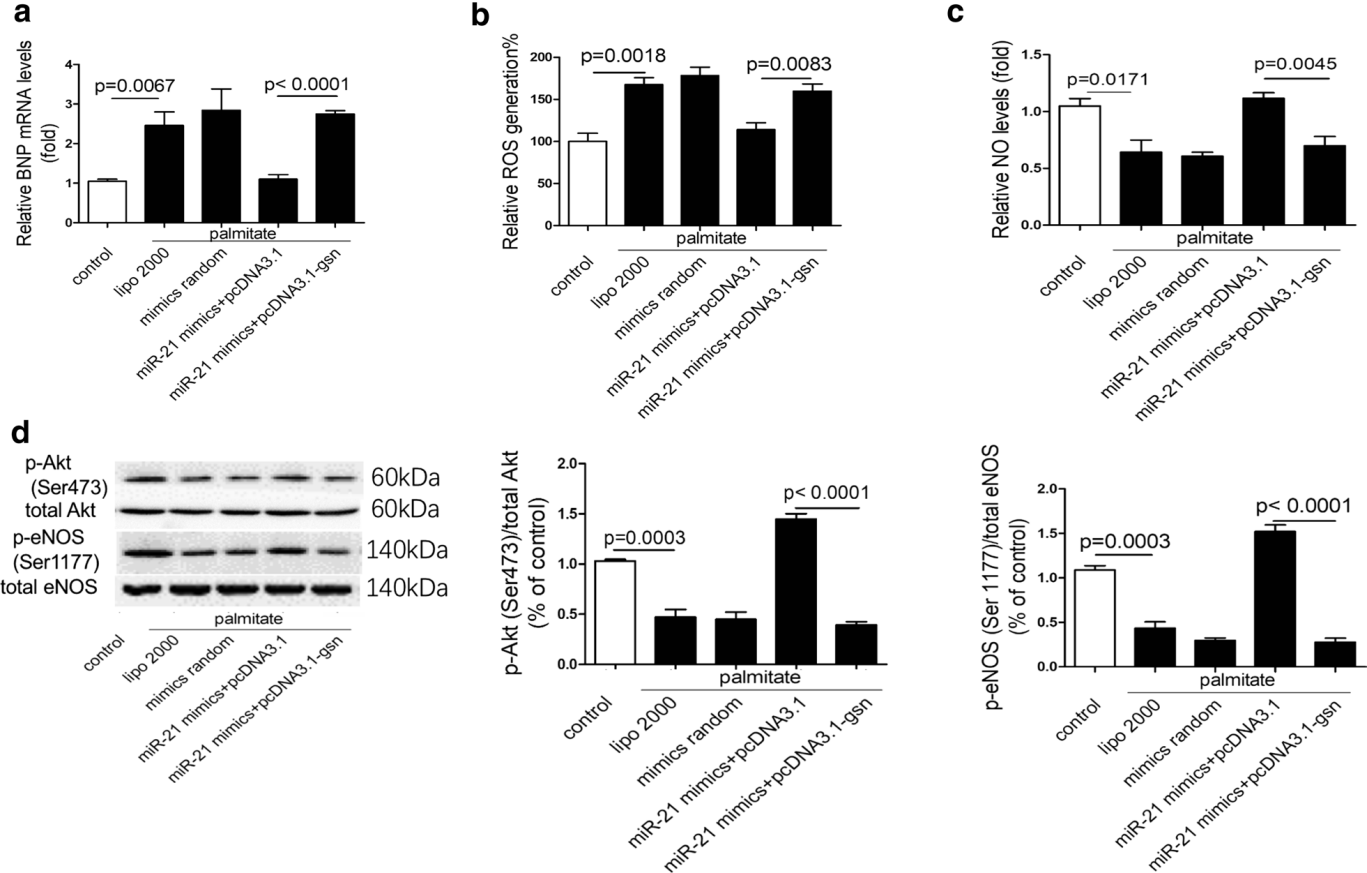
MiR-21 alleviates palmitate-induced injury via GSN. **a** Relative expression levels of BNP in treated H9c2 cells. **b** ROS generation in treated H9c2 cells. **c** Relative NO levels in treated H9c2 cells. **d** Protein levels of p-Akt(Ser473) and p-eNOS(Ser1177) in treated H9c2 cells

We next investigated whether miR-21 regulated cardiac hypertrophy, ROS production and NO levels via GSN pathway. Interestingly, the protective effects of miR-21 were completely blocked by GSN re-expression (Figs. 7g, 8a–d).

In conclusion, our data suggested that miR-21 attenuated palmitate-induced injury by down-regulating GSN expression.

## Discussion

**Fig. 9:**
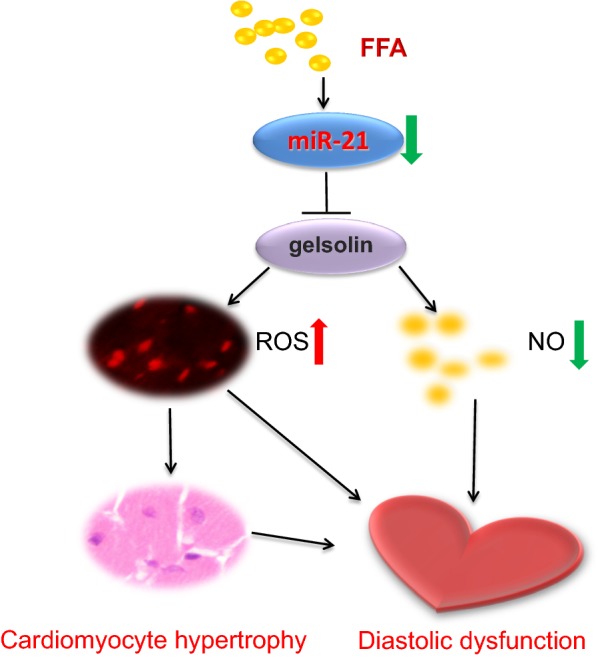
Schematic representation of the association among miR-21, GSN, and diastolic dysfunction in diabetic cardiomyopathy. In db/db mice, decreased miR-21 resulted in GSN overexpression, which increased ROS, decreased NO production, and enhanced cardiac hypertrophy, which finally impaired cardiac diastolic dysfunction

In the present study, we observed down-regulated cardiac miR-21 in db/db mice, which contributed to diabetic cardiomyopathy. We found that miR-21 was able to suppress GSN, an important transcriptional cofactor in signal transduction in cardiovascular diseases. Moreover, the overexpression of exogenous miR-21 efficiently protected against diastolic dysfunction and alleviated cardiac hypertrophy by decreasing ROS and improving NO release via GSN in db/db mice, which suggested a new therapeutic strategy against diabetic cardiomyopathy (Fig. 9).

Previous findings in db/db mice, which were characterized by progressive obese, hyperglycemia and hyperinsulinemia, revealed a strong association between altered myocardial substrate preference and cardiac dysfunction [52–54]. In the current study, db/db mice, an established animal model of diabetic cardiomyopathy, were used to study the pathological process of early diabetic cardiomyopathy [55]. Using hemodynamic examinations, we only observed decreased diastolic function in db/db mice in the absence of measurable contractile dysfunction at the age of 20 weeks, which was consistent with previous data that cardiac diastolic dysfunction was often the earliest hallmark of diabetic cardiomyopathy [4, 5, 56]. Studies revealed that diastolic dysfunction usually occurred in isolation, or prior to systolic dysfunction [8], and diastolic dysfunction was more susceptible to preclinical changes [57].

The pathophysiology of diabetic cardiac dysfunction is complex and multifactorial. Up to now, several mechanisms of diastolic cardiac stiffness have been discovered, including increased myocardial deposition of collagen, myocardial triglyceride accumulation, cardiac hypertrophy, increased generation of ROS, and decreased NO level [3]. In our study, we observed myocardial triglyceride accumulation, cardiac hypertrophy, generation of ROS and decreased NO level in heart from db/db mice. All these factors may initiate diastolic dysfunction, and lead to diabetic cardiomyopathy in the end complicatedly. It is widely accepted that the systolic dysfunction which might occurred in the middle or late stage of diabetic cardiomyopathy is associated with apoptosis and fibrosis. On the other hand, many studies have reported that compared with wild type mice, db/db mice may exhibit no significant fibrosis at 12, 24, and 36 weeks old [58, 59]. In this study, systolic dysfunction and cardiac fibrosis of db/db mice were not observed, which matched with the early phase of diabetic cardiomyopathy, represented by diastolic dysfunction only. As diabetes progresses, there might be fibrosis in the middle or late stage of diabetic cardiomyopathy. Thus, therapies during the early stages of diabetes can potentially delay or impede the progression of various permanent sequelae.

There are multiple pathophysiologic triggers of diabetic cardiomyopathy. Hyperglycemia is one of the central drivers of the metabolic, functional and structural alterations present in the diabetic heart. Whereas FFA is the primary energy source for heart, its level is also elevated in T2DM. High circulating and cellular FFAs can directly elevate peripheral insulin resistance, stimulate apoptosis and trigger a harmful build-up of toxic intermediates, which result in lipotoxicity. These deleterious effects can contribute to impaired cardiac function and adverse remodeling in the diabetic myocardium. The diabetic heart is also characterized by inefficient utilization of glucose for energy production. Studies have reported that FFAs could inhibit glucose oxidation and enhance mitochondrial fatty acid uptake [66, 67]. As cardiac glucose oxidation decreases, cardiac energy provision is almost exclusively via β-oxidation of fatty acids. So, lipotoxicity is thought to be a central player in diabetic heart by promoting acidosis and generating free radicals, inducing ceramide, damaging the mitochondrial membrane, finally making the diabetic heart more prone to injury [68]. Many studies focused on the effects of palmitate in diabetic cardiomyopathy [23, 24]. Studies found that the confounding effects of classical risk factors coexisting in diabetes, such as hyperglycemia, insulin resistance, hyperlipidemia, metabolic disturbances and neurohormonal activation, combination with other risk factors might promote diabetic cardiomyopathy progression. It was reported that patients with type 2 diabetes, metabolic syndrome or obesity all accumulated excess intramyocardial lipid and exhibited systolic or diastolic cardiac dysfunction [3, 69, 70]. In our study, we found that glucose alone did not decrease miR-21 level, whereas palmitate decreased miR-21 level, so we focused on the effects of palmitate in the further study. Moreover, we found palmitate plus glucose together further decreased the expression of miR-21 and aggravated the damages induced by palmitate alone, which stimulate deeper investigations in the future.

MiR-21 is crucial in a number of biological functions and diseases, including development, cancer, cardiovascular diseases and inflammation [60]. In previous study, we found that miR-21 had a positive function in mitochondrial translation, which was sufficient to reduce blood pressure and alleviate cardiac hypertrophy in spontaneous hypertension rats [39]. Studies have reported and many miRNAs involved in mitochondrial dynamics and function in diabetic complications [61]. Here, we observed decreased miR-21 level in both db/db mice and high fat diet fed mice, and miR-21 played a protective role against diabetic cardiomyopathy. In vitro, we found that miR-21 was downregulated in palmitate treated H9c2 cells, while overexpression of miR-21 attenuated the injuries induced by palmitate, suggesting a protective role in cardiomyocyte. While it has been reported that miR-21 was upregulated in fibroblasts with high glucose treatment and exerted its harmful effects [35]. It is an interesting issue that miR-21 plays different roles in different cell types. Deepak et al. found that upon left ventricular pressure overload, cardiac function was only preserved in mice with miR-21 deficiency in nonmyocyte cardiac cells, but not in mice with global or cardiac myocyte-specific ablation [37]. Although in vitro study discovered miR-21 induced damages in high glucose treated fibroblasts [35], in vivo studies showed that miR-21 deficiency in fibroblasts or upregulation of miR-21 in cardiac myocytes protected cardiac function in different pathological condition [34, 37]. In the current study, AAV9 system which provided high-level, stable expression in heart, combined with cardiac specific cTNT promoter was used [62]. Studies have found that this system drove high levels of expression of target gene in the myocardium, compared with other organs [63–65]. Our data demonstrated that miR-21 exerted its protective role directly in cardiac myocytes and encouraged further development of cardiac specific overexpression of miR-21 therapy toward cellular tropism.

Moreover, we verified GSN as a direct target of miR-21. The sequences of gelsolin are not highly conserved among different species; thus, we verified the binding site of miR-21 on gelsolin mRNA in human, mouse and rat cardiomyocytes by RIP. Though we identified GSN as an important target of miR-21 in diabetic cardiomyopathy, there may be still other targets of miR-21.

The expression levels of GSN in human heart tissues and mouse models were increased by different types of cardiac injuries, including pressure overload, acute myocardial infarction, dilated or ischemic cardiomyopathy, and end-stage heart failure [71–73]. Our data showed that GSN was also upregulated in db/db mice, and siRNA against GSN alleviated cardiac damages, while overexpression of GSN showed opposite effects. This was consistent with the previous studies that overexpression of GSN in H9c2 induced cardiac hypertrophy [74]. Although some studies found that the gsn^–/–^ animals did not show significant cardiac hypertrophy or myocyte hypertrophy, gelsolin deficiency improved cardiac systolic function [75]. Interestingly, in this study, we also found that miR-21 was sufficient to alleviate ROS via decreasing GSN which was consistent with previous studies that overexpression of GSN increased ROS level [76, 77]. Li et al. found that the phosphorylation of Akt at Ser473 was significantly increased in gsn^−/−^ mice, suggesting that GSN may inhibit cell survival [72]. Interestingly, we found that in diabetic condition, si-GSN could increase NO level by increasing p-Akt(Ser473) and p-eNOS(Ser1177). Activation of the phosphoinositide-dependent 3 kinase (PI-3K)/Akt axis led to eNOS phosphorylation at Ser1177 and enhanced NO production, which constituted a major protective role in ischemia/reperfusion injury and end-stage heart failure [78–80]. Improvement of Akt-dependent eNOS activity and restoration of Akt-eNOS-NO signaling could attenuate myocardial dysfunction and diabetic cardiomyopathy [81]. We speculated that GSN reduced NO production via Akt-eNOS-NO signaling in diabetic cardiomyopathy induced-diastolic dysfunction.

## Conclusion

Together, here we found that miR-21 improved diastolic function, which was the earliest hallmark of diabetic cardiomyopathy, and alleviated cardiac hypertrophy by reducing elevated ROS and enhancing NO production via GSN. Our data suggested that miR-21 may be a promising therapeutic target for treating diabetic cardiomyopathy.

## Additional file

**Additional file 1.** Additional figures and tables.

## Abbreviations

ROS: reactive oxygen species
NO: nitric oxide
miRNA: microRNA
eNOS: endothelial nitric oxide synthase
FA: fatty acid
FBS: fetal bovine serum
rAAV: recombinant adeno-associated virus
TUNEL: terminal dexynucleotidyl transferase (TdT)-mediated dUTP nick end labelling
TC: total cholesterol
TG: triglyceride
LDL: low-density lipoprotein
HDL: high-density lipoprotein
ANP: atrial natriuretic peptide
BNP: brain natriuretic peptide
UTR: untranslated region
