# Supplementary material for: MiR-21 protected against diabetic cardiomyopathy induced diastolic dysfunction by targeting gelsolin
Source: Cardiovasc Diabetol. 2018 Sep 4;17:123. doi: 10.1186/s12933-018-0767-z (PMC6122727; doi:10.1186/s12933-018-0767-z)
Supplement: Supplementary file 1 — Additional file 1. Additional figures and tables. [file 12933_2018_767_MOESM1_ESM.doc]

**Additional file 1: Figure S1. The validation of isolated primary cardiomyocyte.**

A

**
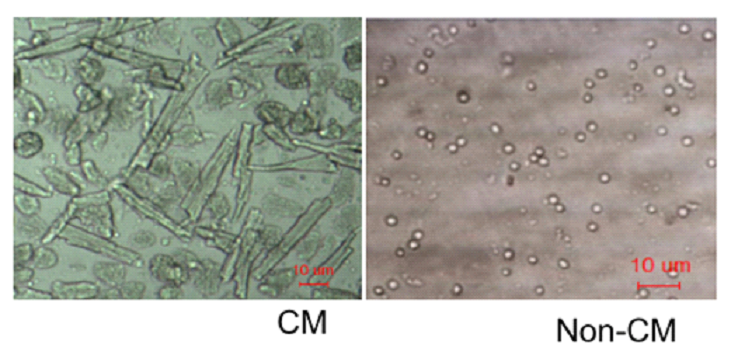
**

**B**

**
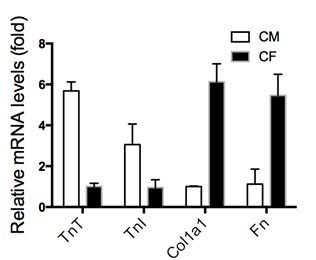
**

**Additional file 1: Figure S1. The validation of isolated primary cardiomyocyte.** (A) The morphology of isolated cells. (B) The expression of cell type markers in isolated cells.

**Additional file 1: Figure S2. The validation of rAAV delivery system.**

**A**


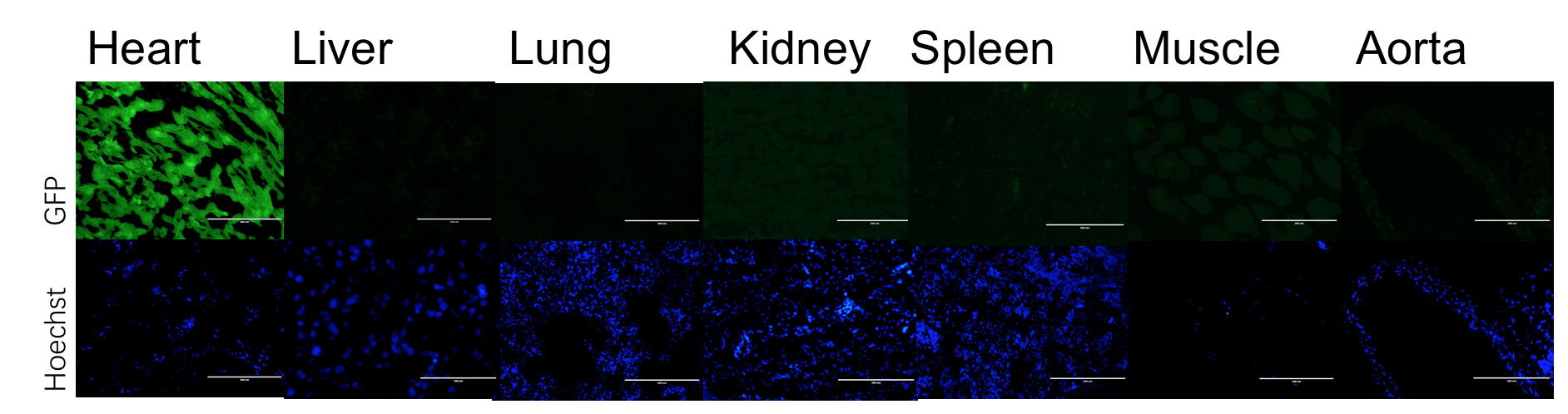


**B**


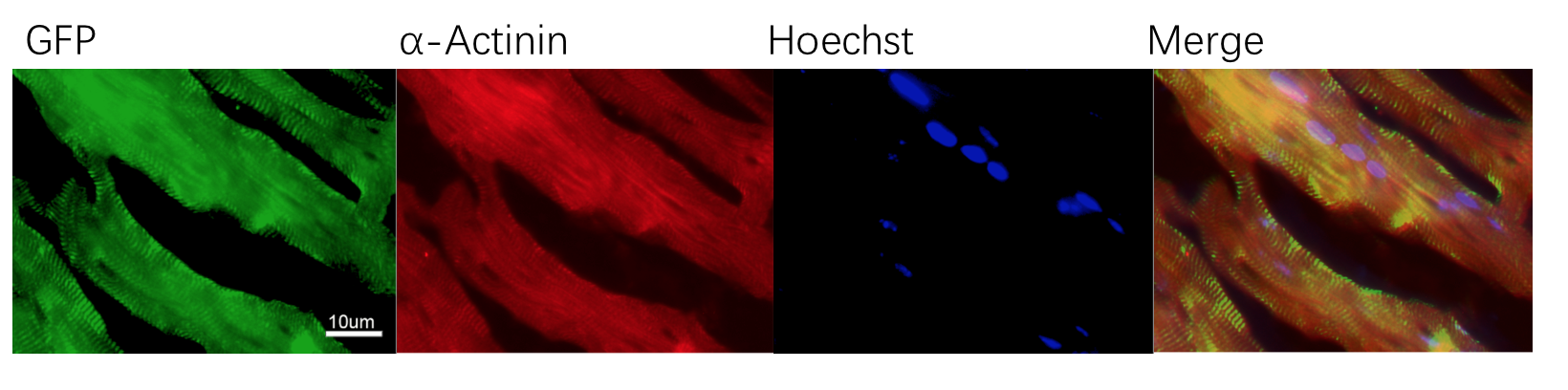


**C**


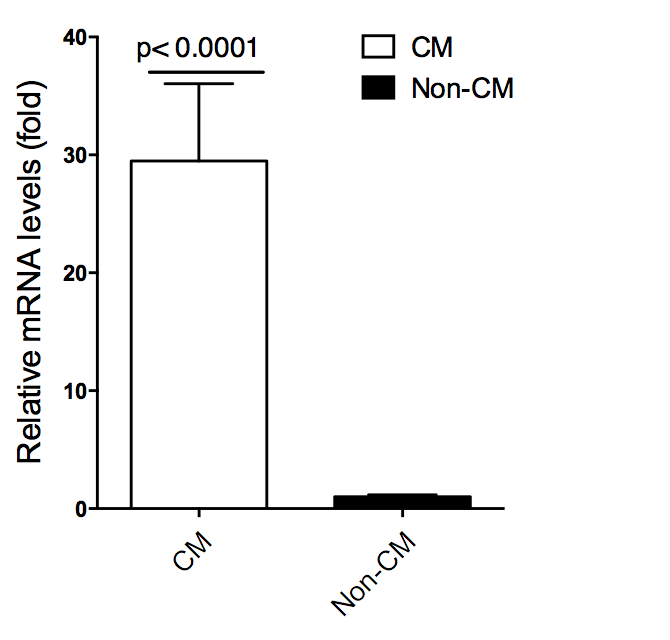


**Additional file 1: Figure S2. The validation of rAAV delivery system.** (A) In situ detection of GFP in different organs from treated mice. (B) In situ detection of GFP in heart from treated mice. (C) Detection of GFP mRNA level in isolated primary cardiomyocytes from treated mice.

**Additional file 1: Figure S3. Cardiac function analysis of treated wt control and db/db mice.**


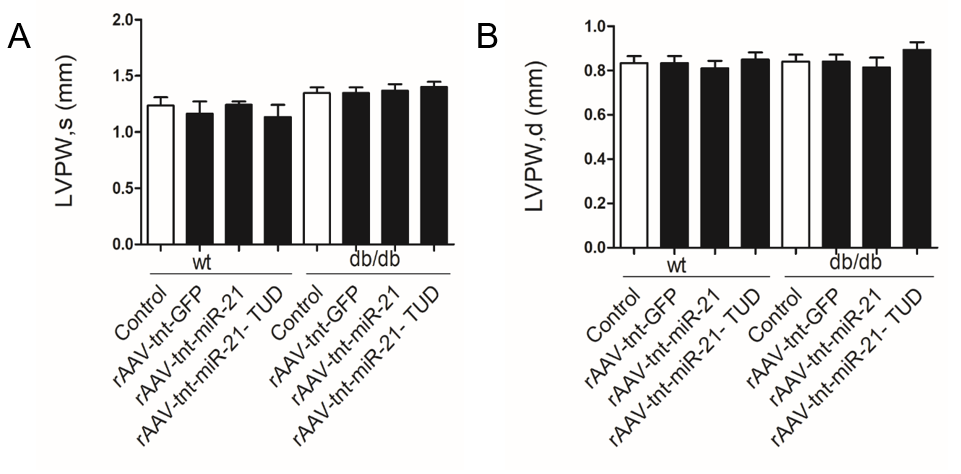


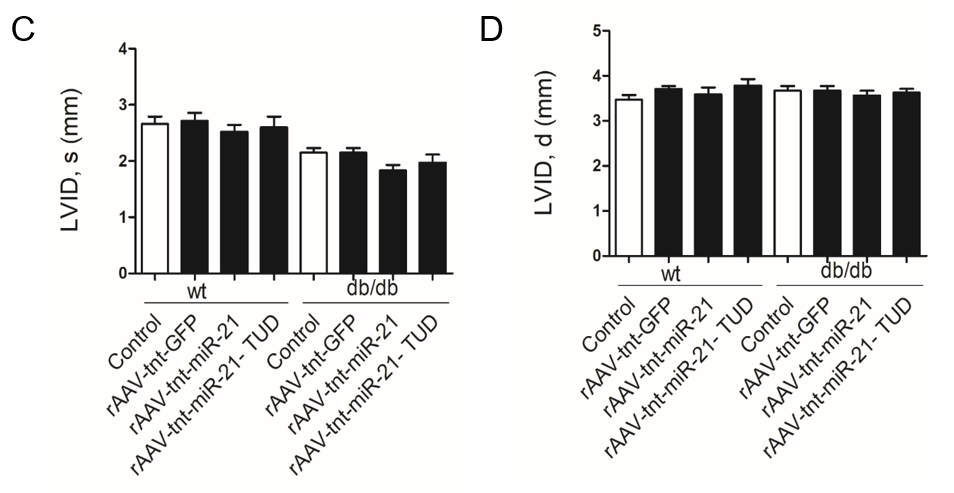


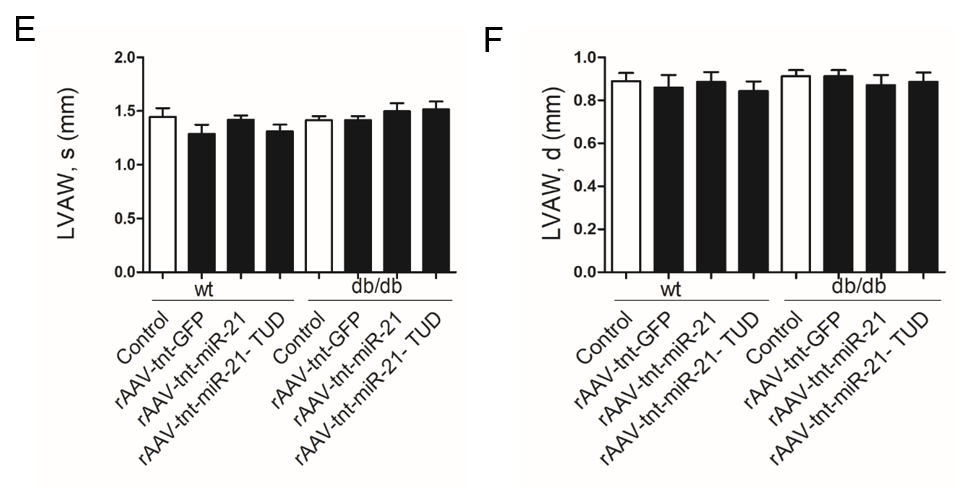


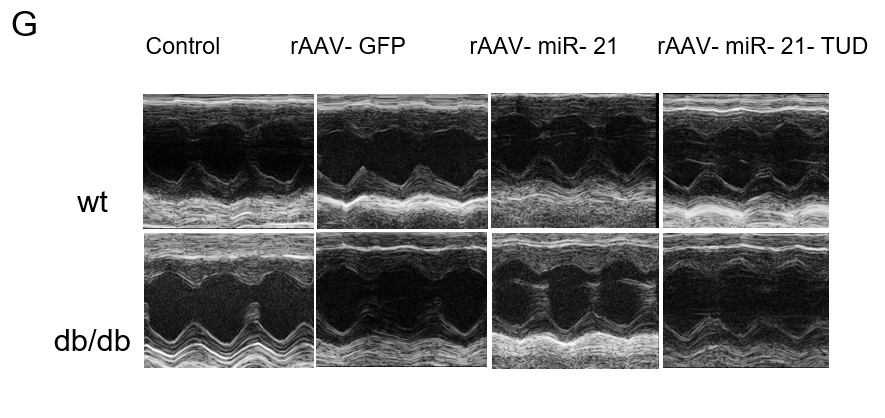


**
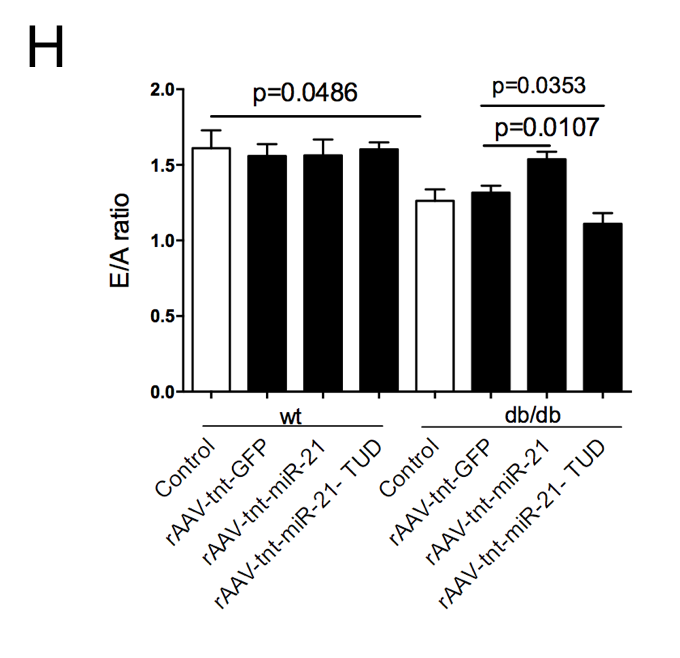
**

**Additional file 1: Figure S3. Cardiac function analysis of treated db/db mice and wt controls.** (A-F) LVPW(s), LVPW(d), LVID(s), LVID(d), LVAW(s) and LVAW(d) were quantitatively analyzed. (G) The original images of echocardiography. (H) E/A ratio of C57BL/Ks controls and db/db mice.

**Additional file 1: Figure S4.** **Cardiac overexpression or inhibition of miR-21 had no effects on metabolic characteristics.**


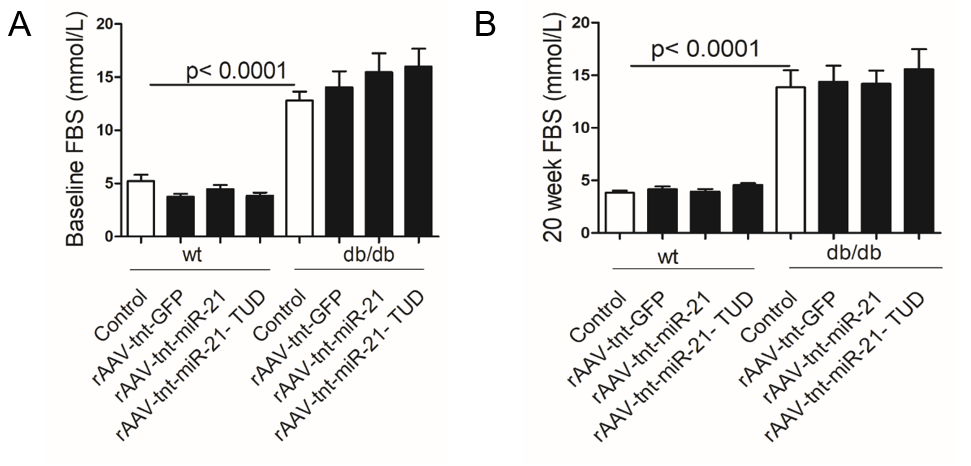


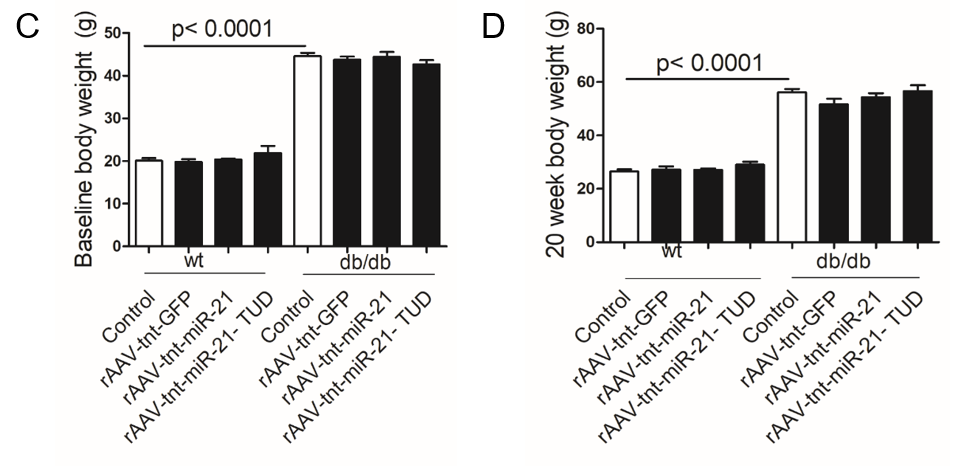


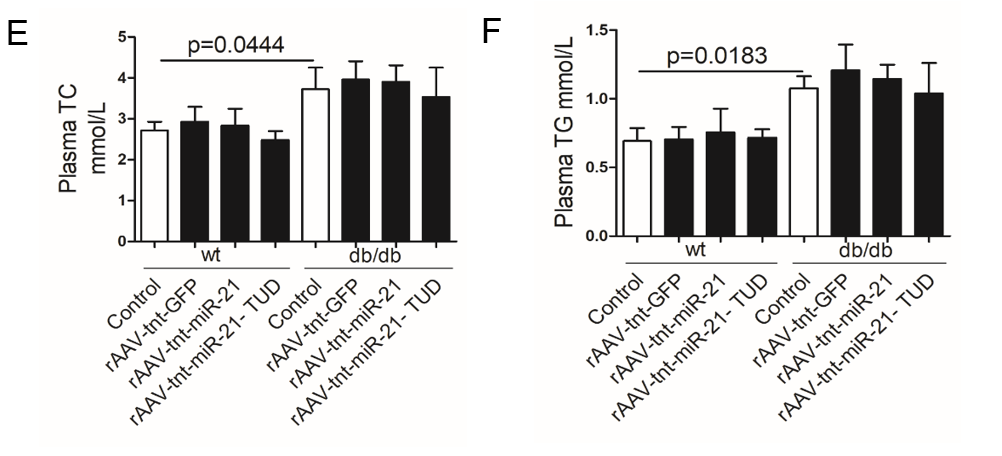


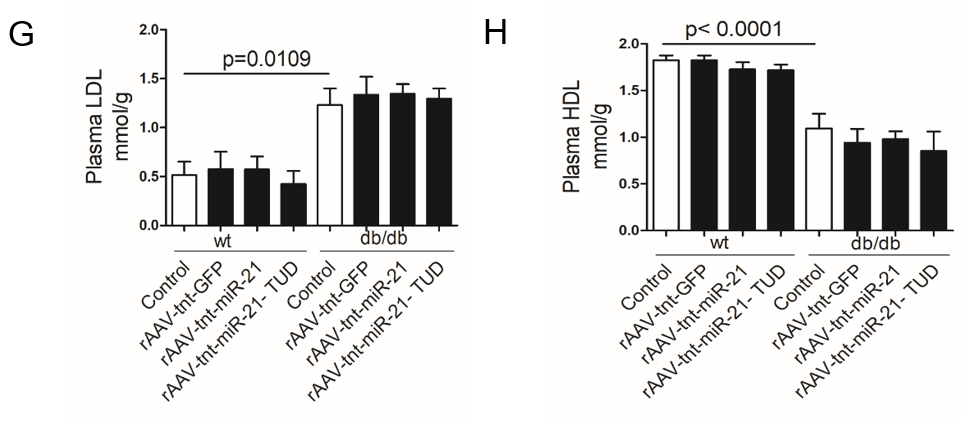


**Additional file 1: Figure S4.** **Cardiac overexpression or inhibitor of miR-21 had no effects on metabolic characteristics.** (A-D) Fasting glucose and body weight in treated mice. (E-H) Plasma TC, LDL, HDL and TG in treated mice.

**Additional file 1: Figure S5.** **Histomorphology detection in treated mice.**


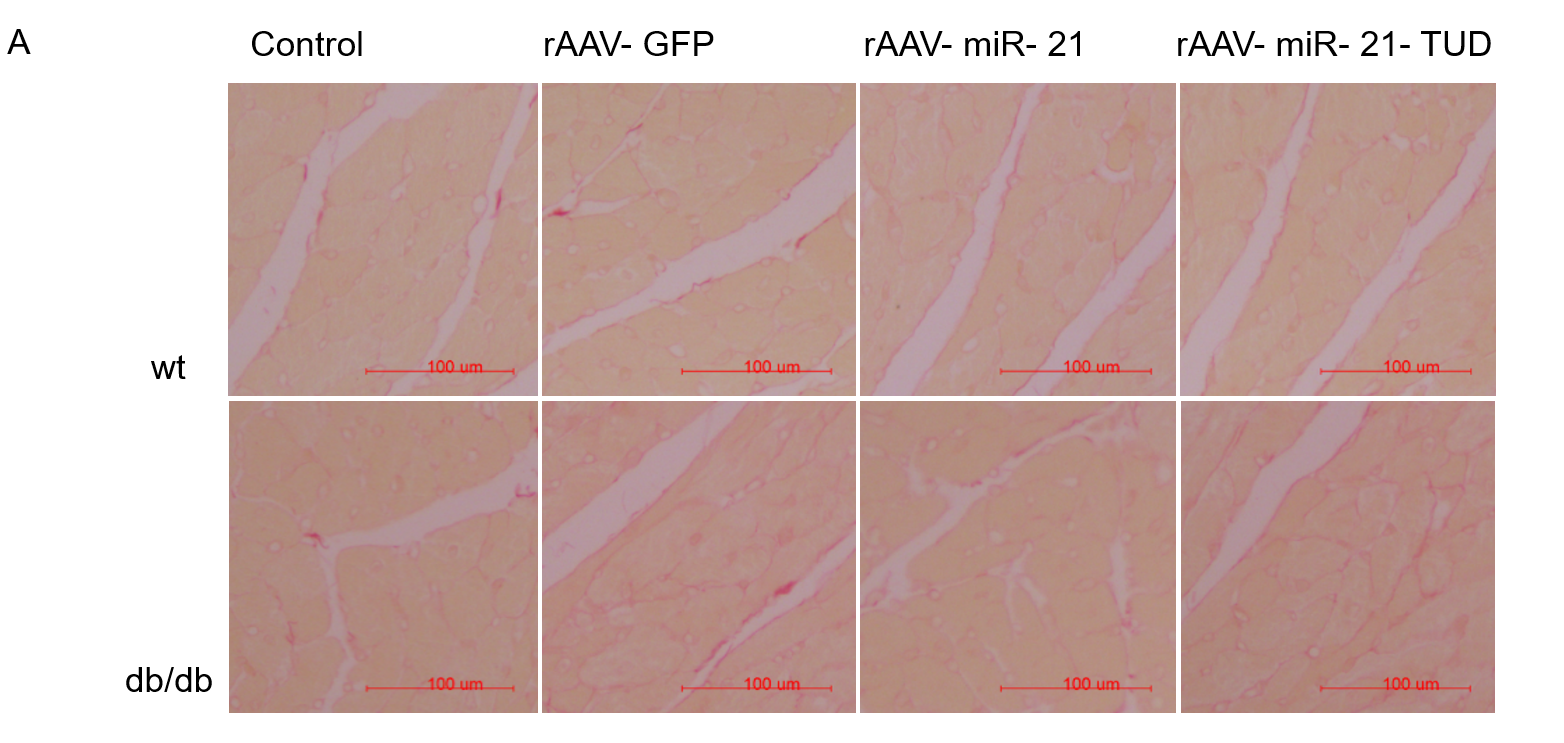


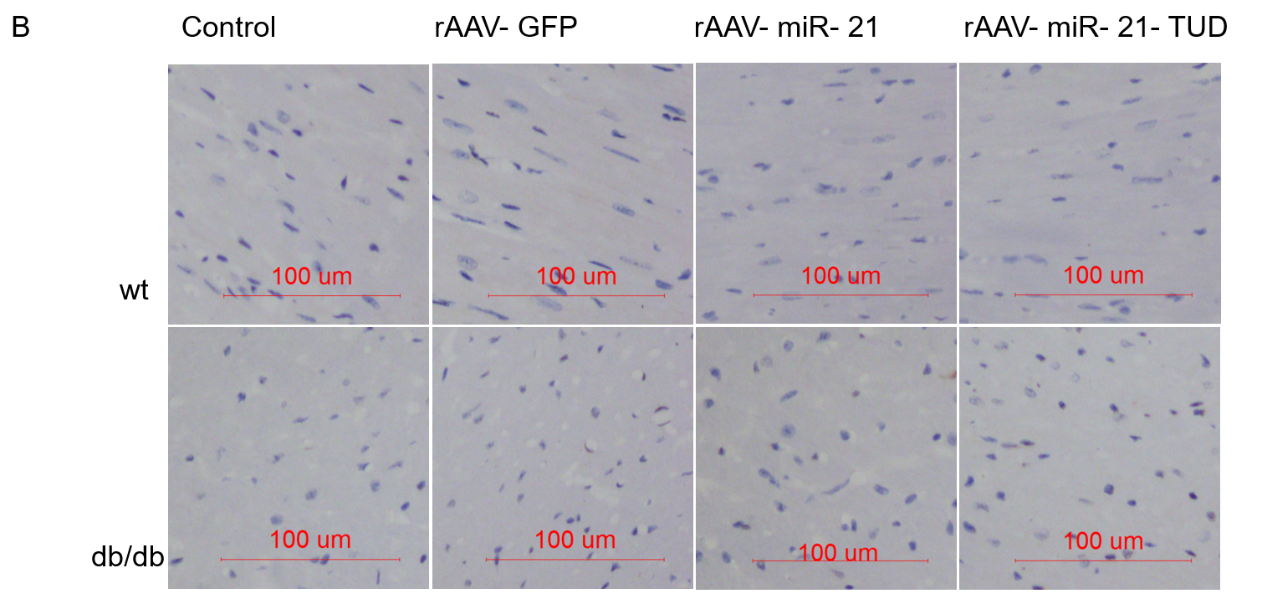


C

**
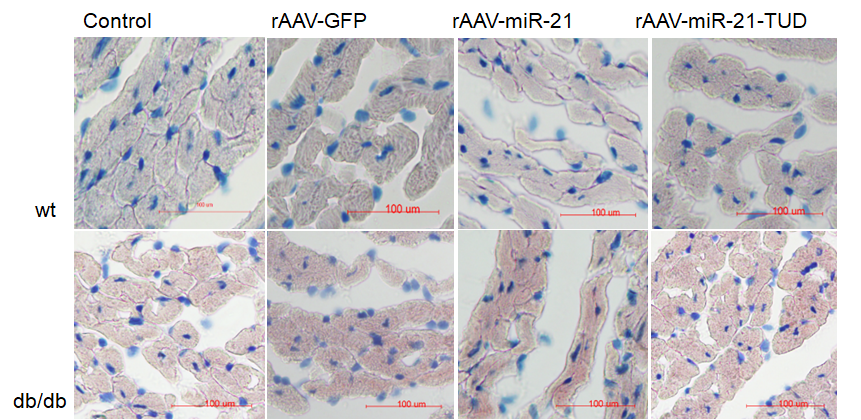
**


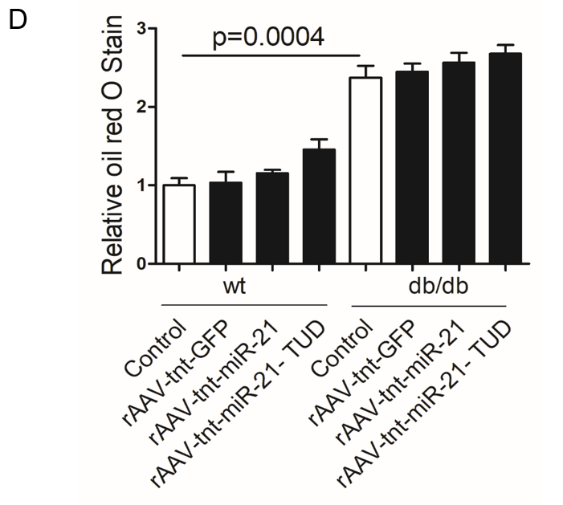


**Additional file 1: Figure S5.** **Histomorphology detection in treated mice.** (A) Representative images of myocardial fibrosis detected by Sirius Red Staining in treated mice. (B) Representative images of apoptosis detected by TUNEL staining in treated mice. (C and D) Histological analysis of Oil Red staining in treated mice.

**Additional file 1: Figure S6.** **MiR-21 did not change the expression of p-eNOS (Thr495) or iNOS in vivo.**

**
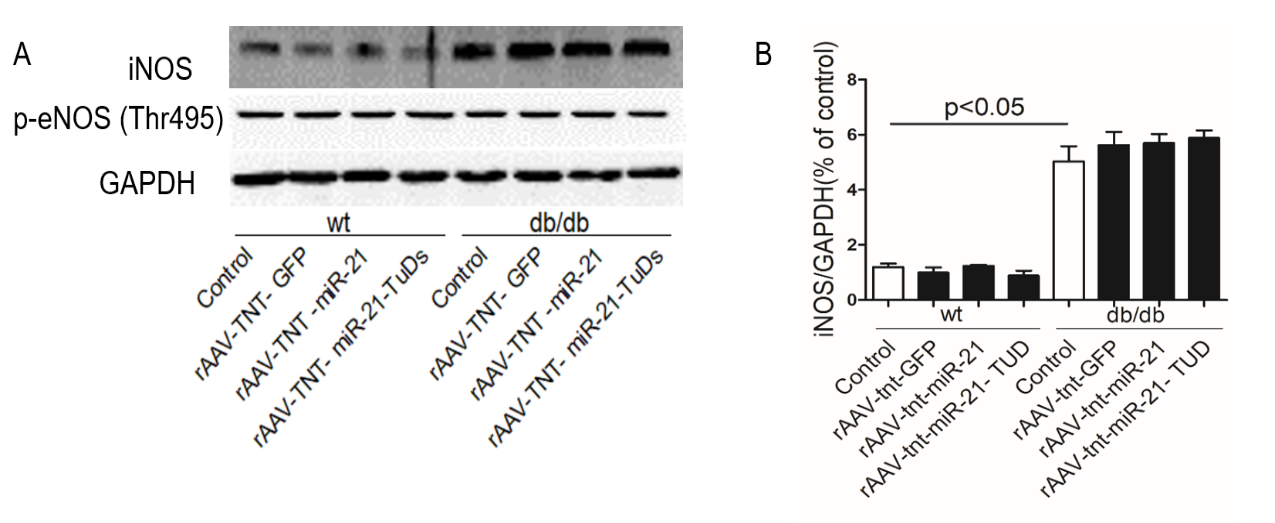
**


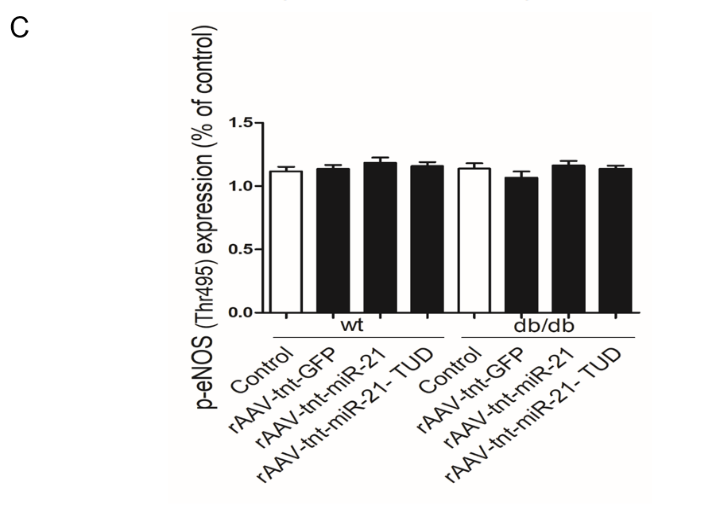


**Additional file 1: Figure S6.** **MiR-21 did not change the expression of p-eNOS (Thr495) or iNOS.** (A-C) Protein levels of p-eNOS (Thr495) and iNOS in heart of treated mice.


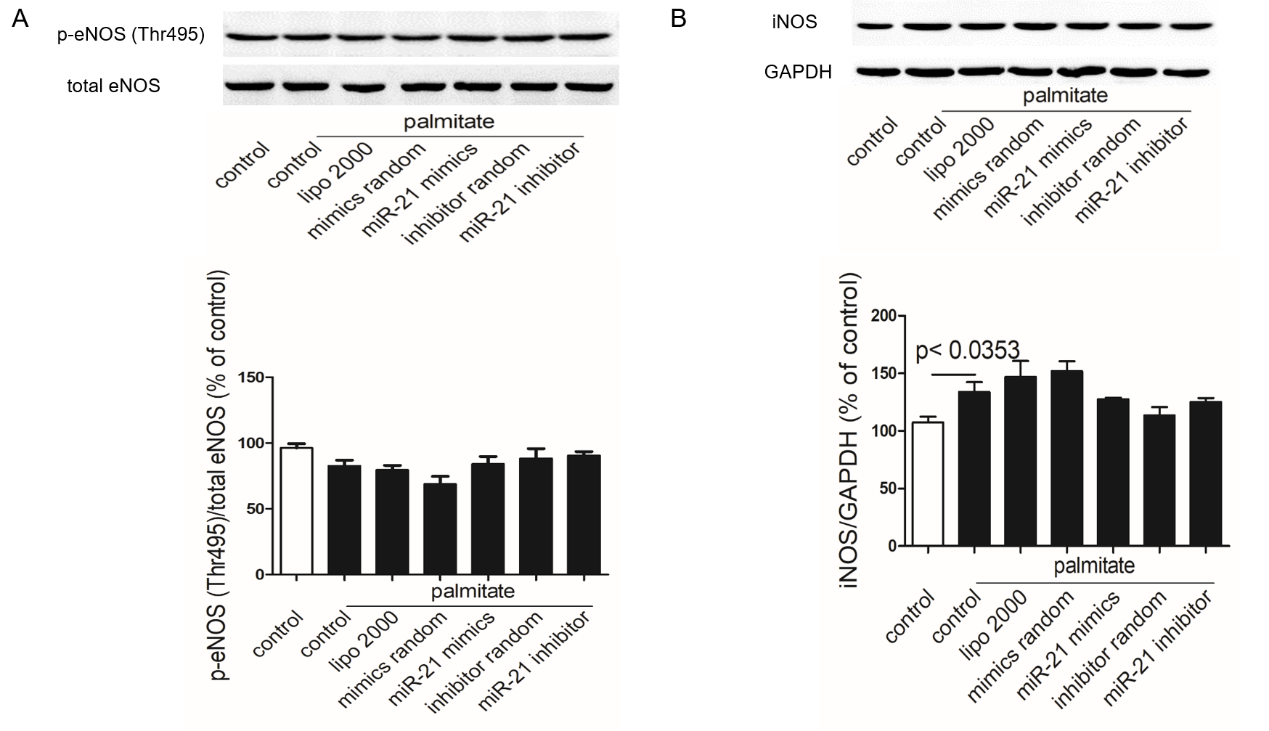
**Additional file 1: Figure S7. MiR-21 did not change the expression of p-eNOS (Thr495) or iNOS in vitro.**

**Additional file 1: Figure S7. MiR-21 did not change the expression of p-eNOS (Thr495) or iNOS in vitro.** (A-B) Protein levels of p-eNOS (Thr495) and iNOS in H9c2 cells.


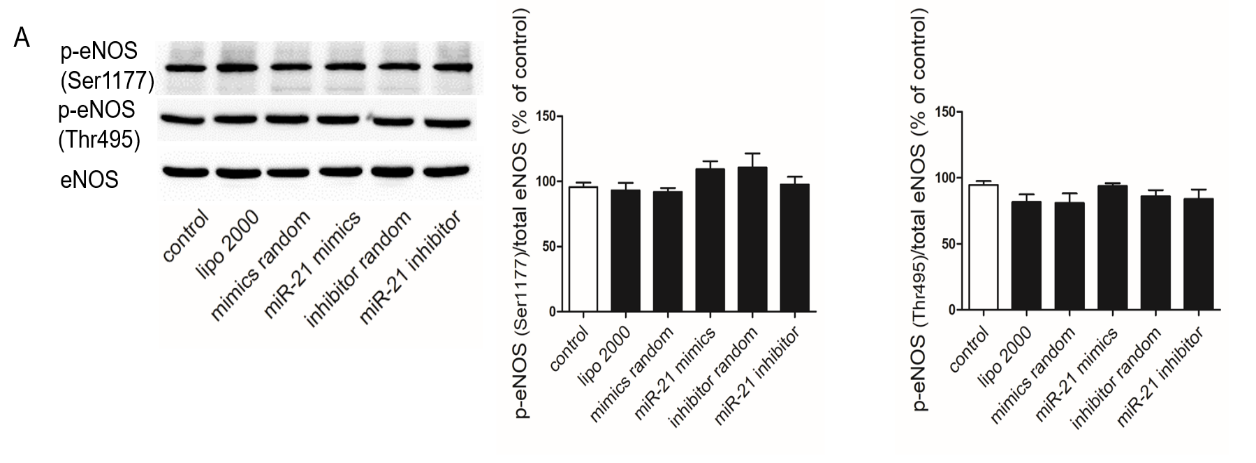
**Additional file 1: Figure S8. In physiological status, miR-21 had no effect on NOS.**

**
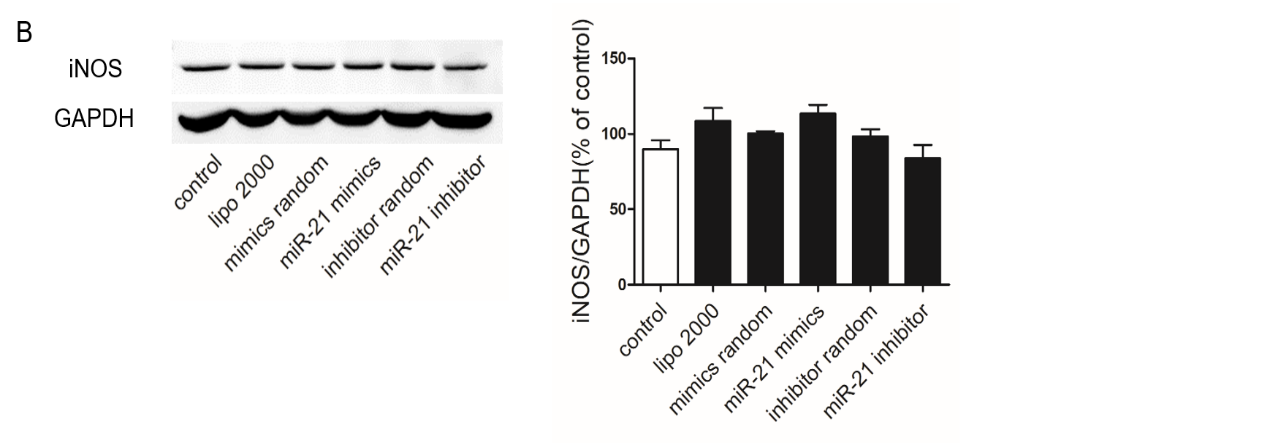
**

**Additional file 1: Figure S8. In physiological status, miR-21 had no effect on NOS.** (A) Protein levels of p-eNOS (Ser1177) and p-eNOS (Thr495) in H9c2 cells. (B) Protein levels of iNOS in H9c2 cells.

**Additional file 1: Figure S9. MiR-21 protected against diabetic condition induced cardiomyocyte hypertrophy in human cardiomyocytes.**

**
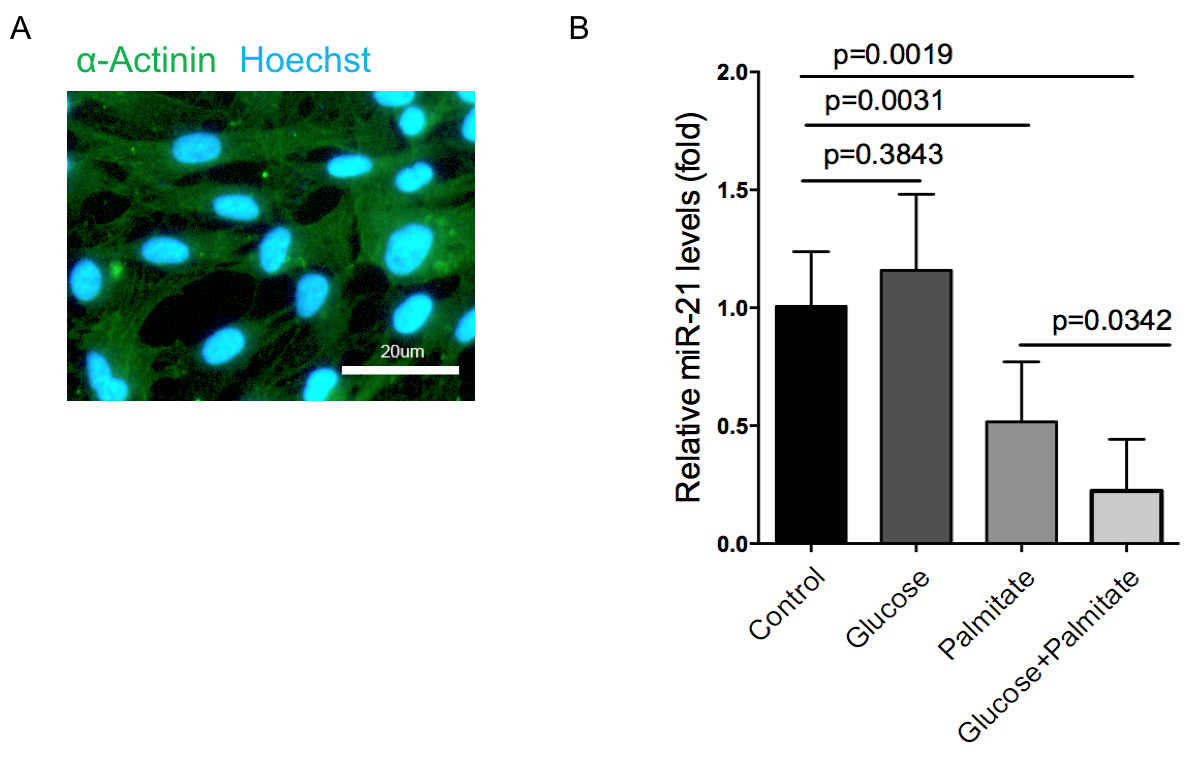
**

**
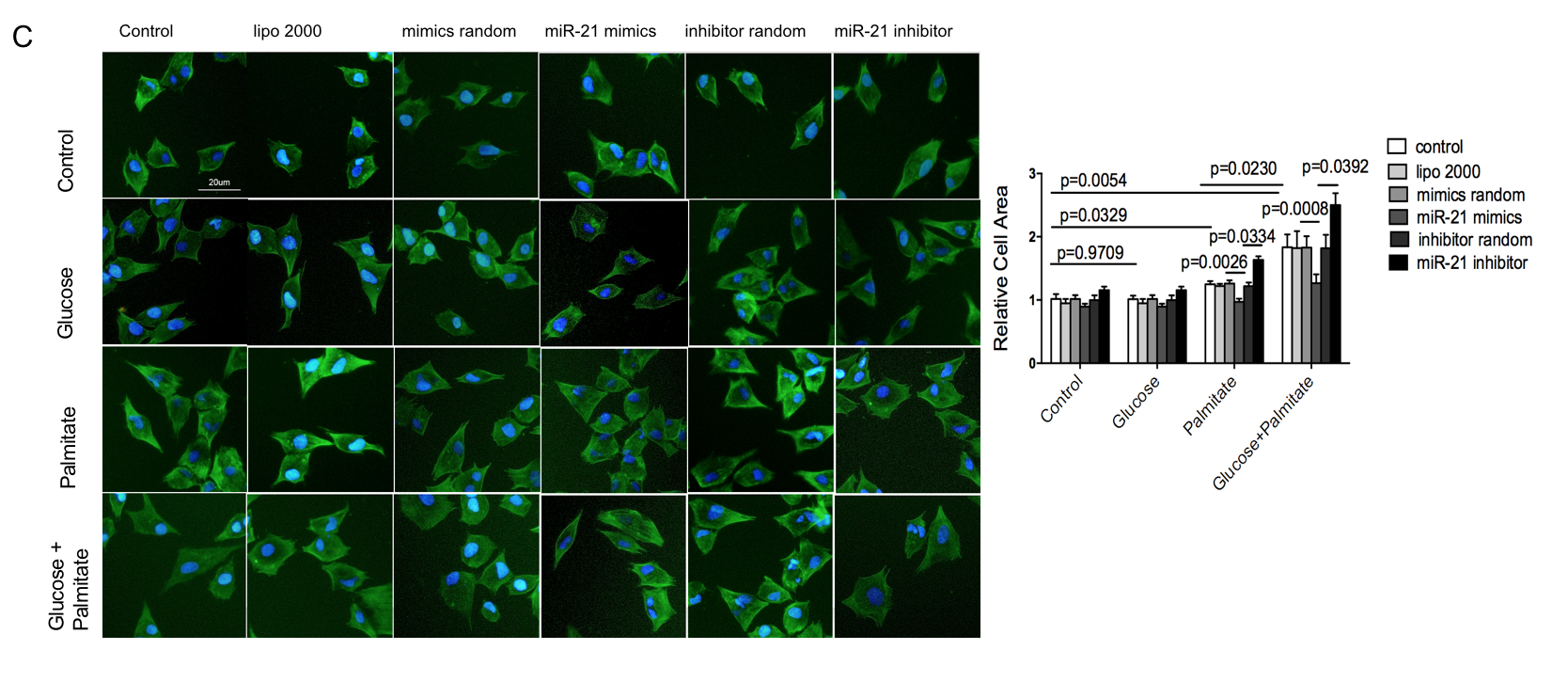
**

**
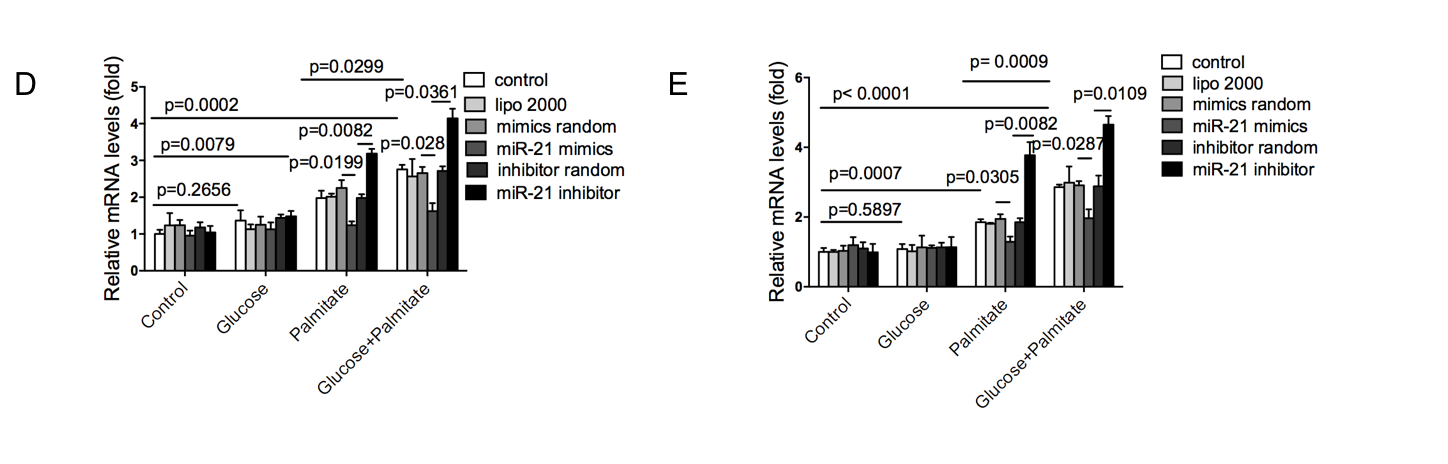
**

**
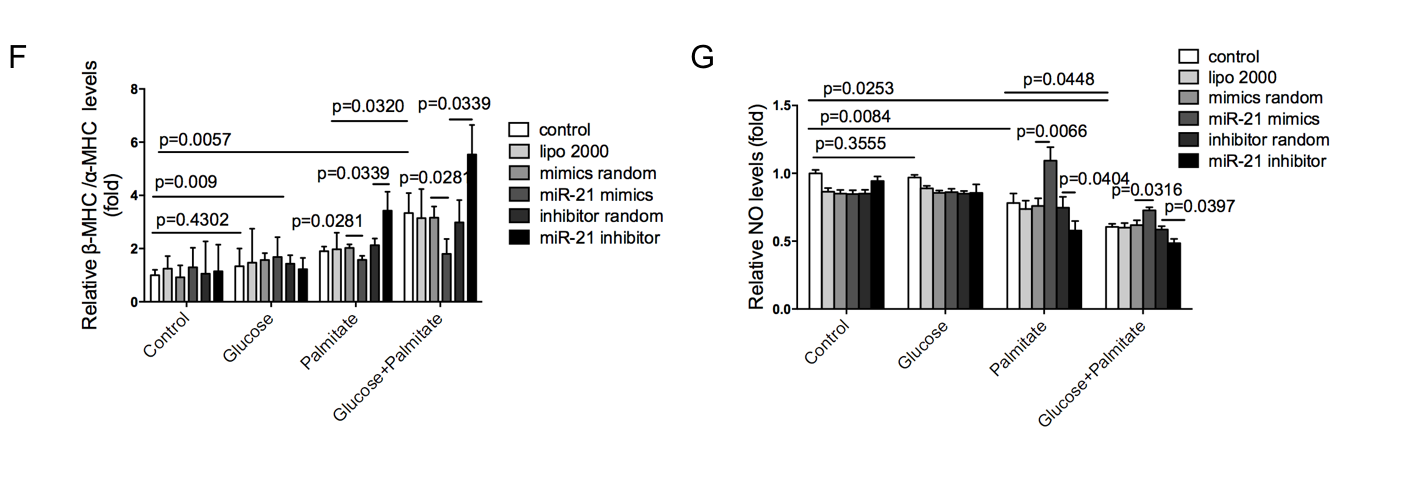
**

**Additional file 1: Figure S9. MiR-21 protected against diabetic condition induced cardiomyocyte hypertrophy in human cardiomyocytes.** (A) Representative images of immunohistochemical staining for sarcomeric a-actinin. (B) Relative expression of miR-21 in treated cells. (C) Representative images and relative cell area determined by quantitation analysis of cardiomyocyte by Phalloidin staining. (D-F) Relative expression levels of ANP, BNP, and β-MHC/α-MHC in treated cells. (G) Relative NO levels in treated cells.

**Additional file 1: Figure S10. Potential targets of miR-21.**

**
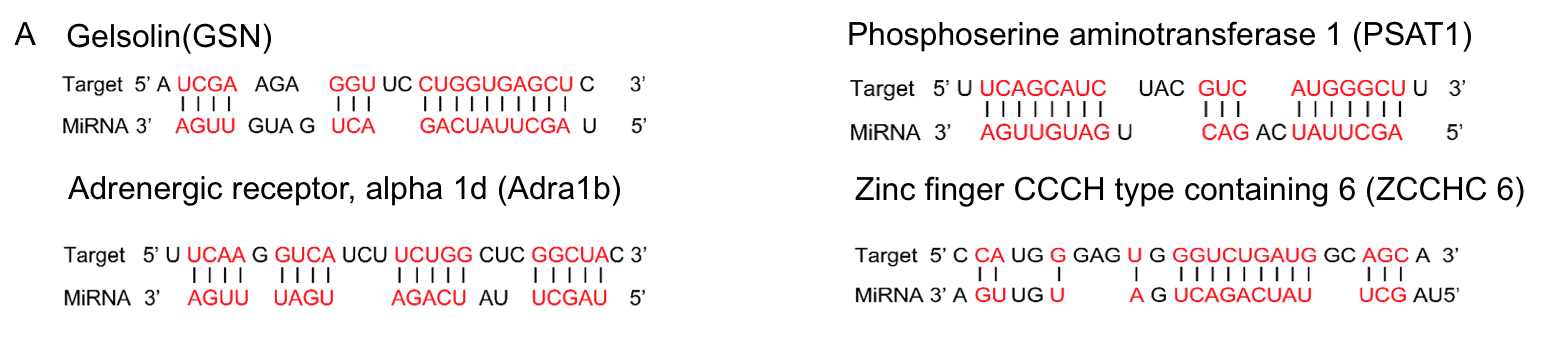
**

**Additional file 1: Figure S10. Potential targets of miR-21.** (A) Representative images of potential targets of miR-21.

**Additional file 1: Table S1. Sequences and sources of PCR primers.**

| **Primer name** | **Sequence (5'-3')** | **Company** |
| --- | --- | --- |
| rANP-F | GAGCAAATCCCGTATACAGTGC | Tianyihuiyuan gene |
| rANP-R | ATCTTCTACCGGCATCTTCTCC | Tianyihuiyuan gene |
| rBNP-F | GCTGCTGGAGCTGATAAGAGAA | Tianyihuiyuan gene |
| rBNP-R | GTTCTTTTGTAGGGCCTTGGTC | Tianyihuiyuan gene |
| rα-MHC-F | AAGGTGAAGGCCTACAAGCG | Tianyihuiyuan gene |
| rα-MHC-R | TATTGTGGGATAGCAACAGCGA | Tianyihuiyuan gene |
| rβ-MHC-F | ACCTGTCCAAGTTCCGCAAG | Tianyihuiyuan gene |
| rβ-MHC-R | TGGAGCTGGGTAGCACAAG | Tianyihuiyuan gene |
| rGAPDH-F | TGTGAACGGATTTGGCCGTA | Tianyihuiyuan gene |
| rGAPDH-R | GATGGTGATGGGTTTCCCGT | Tianyihuiyuan gene |
| mANP-F | AGGCAGTCGATTCTGCTTGA | Tianyihuiyuan gene |
| mANP-R | CGTGATAGATGAAGGCAGGAAG | Tianyihuiyuan gene |
| mBNP-F | TAGCCAGTCTCCAGAGCAATTC | Tianyihuiyuan gene |
| mBNP-R | TTGGTCCTTCAAGAGCTGTCTC | Tianyihuiyuan gene |
| mα-MHC-F | CTGTCCAAGTTCCGCAAGGT | Tianyihuiyuan gene |
| mα-MHC-R | TCGTGCATCTTCTTGGCACC | Tianyihuiyuan gene |
| mβ-MHC-F | AGCCTCAGCAGAGGAGTACA | Tianyihuiyuan gene |
| mβ-MHC-R | GGCTGAGCCTTGGATTCTCA | Tianyihuiyuan gene |
| mGAPDH-F | GACCTCATGGCCTACATGGC | Tianyihuiyuan gene |
| mGAPDH-R | ATTATGGGGGTCTGGGATGGA | Tianyihuiyuan gene |
| hANP-F | GGATTGCTCCTTGACGACG | Tianyihuiyuan gene |
| hANP-R | GGGCACGACCTCATCTTCTAA | Tianyihuiyuan gene |
| hBNP-F | CTTTGGGAGGAAGATGGAC | Tianyihuiyuan gene |
| hBNP-R | GTGGAATCAGAAGCAGGTGT | Tianyihuiyuan gene |
| hα-MHC-F | CTCCGCAAGTCAGAAGGAGC | Tianyihuiyuan gene |
| hα-MHC-R | CACTTCCTCCTGGTCCACAACG | Tianyihuiyuan gene |
| hβ-MHC-F | GACACACTTGAGTAGCCCAGG | Tianyihuiyuan gene |
| hβ-MHC-R | GAACTTGGGTGGGTTCTGCT | Tianyihuiyuan gene |
| hGAPDH-F | AGAAGGCTGGGGCTCATTTG | Tianyihuiyuan gene |
| hGAPDH-R | AGGGGCCATCCACAGTCTTC | Tianyihuiyuan gene |
| rGSN-F | AGACTGCCTACCGCACATCC | Tianyihuiyuan gene |
| rGSN-R | CCTTTCCAACCCAGACAAAGA | Tianyihuiyuan gene |
| mGSN-F | AGCATGTGCAGGTGGAAGAAG | Tianyihuiyuan gene |
| mGSN-R | TGGAGCAGGCAAAGAGTCG | Tianyihuiyuan gene |
| hGSN-F | AGGTGTCCAAGGGCATCCGGGA | Tianyihuiyuan gene |
| hGSN-R | CGCATACTCTTGGGGAAGCGGGT | Tianyihuiyuan gene |

**Additional file 1: Table S2. Upregulated genes in db/db heart from mice compared with wt controls.**

| | **Gene symbol** | **Fold Change (db/db vs wt)** | | --- | --- | | HMGCS2 | 20.07 | | AC0T8 | 11.74 | | Kcnk1 | 7.701 | | Cyp2b10 | 5.702 | | hh | 5.232 | | ACOT1 | 5.021 | | Cox6b2 | 4.577 | | GSN | 4.395 | | HMGCS2 | 4.345 | | ZCCHC6 | 4.247 | | ACOT2 | 3.968 | | PTH1R | 3.739 | | CSPG1 | 3.675 | | PTPRD | 3.667 | | DHNS8 | 3.57 | | UCP3 | 3.544 | | EHHADH | 3.333 | | Panx2 | 3.299 | | SDK2 | 3.287 | | FKBP5 | 3.286 | | ABGPTL4 | 3.245 | | Slc17a7 | 3.084 | | CA4 | 2.846 | | THBS2 | 2.623 | | CLDN15 | 2.544 | | Lgals4 | 2.519 | | Cyp4b1 | 2.447 | | HTRA3 | 2.441 | | Cdo1 | 2.437 | | LGR16 | 2.405 | | Ndrg4 | 2.394 | | CYP1B1 | 2.387 | | Atp8a1 | 2.369 | | GNMT | 2.363 | | Asb4 | 2.356 | | FMO3 | 2.337 | | Ptk2b | 2.335 | | CPT1A | 2.334 | | EPOR | 2.332 | | PSAT1 | 2.303 | | Dpep1 | 2.295 | | Acot2 | 2.283 | | Dctn6 | 2.265 | | pigL | 2.264 | | Adra1b | 2.262 | | SLC45A3 | 2.253 | | Tbx5 | 2.242 | | Rps6ka5 | 2.211 | | trdn | 2.209 | | DDX10 | 2.208 | | Sult1a1 | 2.183 | | Rnpc3 | 2.181 | | FBP2 | 2.175 | | Rbp7 | 2.17 | | KLF10 | 2.169 | | TBL1XR1 | 2.155 | | FMO2 | 2.102 | | Thbs2 | 2.095 | | Ptp4a1 | 2.094 | | SBSN | 2.064 | | SAFB | 2.064 | | Slc27a1 | 2.05 | | MAPK10 | 2.048 | | GATA6 | 2.031 | | CYP2A1 | 2.03 | | Slc25a20 | 2.021 | | Dctn6 | 2.017 | | CIPBP | 2.012 | | MLYCD | 2.004 | |
| --- | --- | --- | --- | --- | --- | --- | --- | --- | --- | --- | --- | --- | --- | --- | --- | --- | --- | --- | --- | --- | --- | --- | --- | --- | --- | --- | --- | --- | --- | --- | --- | --- | --- | --- | --- | --- | --- | --- | --- | --- | --- | --- | --- | --- | --- | --- | --- | --- | --- | --- | --- | --- | --- | --- | --- | --- | --- | --- | --- | --- | --- | --- | --- | --- | --- | --- | --- | --- | --- | --- | --- | --- | --- | --- | --- | --- | --- | --- | --- | --- | --- | --- | --- | --- | --- | --- | --- | --- | --- | --- | --- | --- | --- | --- | --- | --- | --- | --- | --- | --- | --- | --- | --- | --- | --- | --- | --- | --- | --- | --- | --- | --- | --- | --- | --- | --- | --- | --- | --- | --- | --- | --- | --- | --- | --- | --- | --- | --- | --- | --- | --- | --- | --- | --- | --- | --- | --- | --- | --- | --- |
